# Alterations of *SLC14A1* mRNA structure are frequently detected in weak Jk^a^ presenting donors

**DOI:** 10.1371/journal.pone.0360437

**Published:** 2026-10-14

**Authors:** Shuang Liang, Yanwen Liang, Fan Wu, Tong Liu, Liyan Sun, Bin Luo, Yu Wang, Yi Liu, Bo Feng, Jianwei Ren

**Affiliations:** 1 Institute of Transfusion Medicine, Shenzhen Blood Center, Shenzhen, Guangdong, People’s Republic of China; 2 Guangdong Provincial Key Laboratory of Research and Development of Natural Drugs, School of Pharmacy, Guangdong Medical University, Zhanjiang, Guangdong, People’s Republic of China; 3 School of Biomedical Sciences, The Chinese University of Hong Kong, Hong Kong, People’s Republic of China; 4 School of Pharmacy, Hangzhou Normal University, Hangzhou, Zhejiang, People’s Republic of China; 5 School of Computer Science, Zhuhai College of Science and Technology, Zhuhai, Guangdong, People’s Republic of China; 6 Centre for Regenerative Medicine and Health, Hong Kong Institute of Science & Innovation, Chinese Academy of Sciences, Hong Kong, People’s Republic of China; Roswell Park Cancer Institute, UNITED STATES OF AMERICA

## Abstract

RNA structural changes caused by mis-splicing or transcription errors may affect antigenic features of cell surface proteins. They are especially noteworthy in the field of blood transfusion where the discrepancy between DNA-based molecular testing and serological blood typing are often encountered in clinical practice and lead to mismatched blood transfusion. In the Kidd (JK) blood group system weak Jk^a^ (Jk(a + ^w^)) phenotype is usually missed in routine serological tests and causes delayed hemolytic transfusion reactions. However molecular bases underlying Jk(a + ^w^) phenotype are still not clear although plentiful genomic DNA-based studies have been conducted. In this study, we used PacBio long-read sequencing to characterize open reading frame (ORF) region of *SLC14A1* mRNA obtained from reticulocytes of 18 blood donors possessing JK*A alleles. Among them, 8 donors were determined as normal Jk^a^ antigen presentation and 10 donors showed Jk(a + ^w^) phenotype in routine serological tests. We found that Jk(a + ^w^) subjects demonstrated different exon combination patterns compared to those individuals presenting normal Jk^a^ antigens, implying the resulted SLC14A1 protein isoforms could make different contributions to the formation of the JK antigenicity. Moreover, exon skipping and exon inclusion that were frequently found in Jk(a + ^w^) individuals could induce frameshift mutations. In addition, high levels of nucleotide insertions or deletions (indels) at *130A* position were detected in the transcripts of Jk(a + ^w^) individuals. Technical artifacts were examined using synthesized RNA templates and the results suggested that artifacts generated in reverse transcription and PCR amplification (RT-PCR) processes were unlikely the causes of these observations. All the results indicate that *SLC14A1* RNA splicing and transcription fidelity play crucial roles in determining Jk^a^ antigen presentation.

## Introduction

Variations in RNA structure, caused by mis-splicing or transcription errors, may lead to a wide range of diseases including neurological diseases [1] and cancers [2]. In addition, it has been reported that changes of RNA structures play noteworthy roles in determining antigenic epitopes of cell surface proteins, such as cancer neoantigens [3] and blood group antigens [4]. The findings are especially significant in interpreting the mechanisms underlying blood transfusion reactions due to blood typing discrepancies in clinical practice [5].

The Kidd blood group system, encoded by the *SLC14A1* gene through its UT-B urea transporter protein, plays a pivotal role in transfusion medicine due to its association with severe delayed hemolytic transfusion reactions (DHTRs) [6,7]. Notably, anti-Jk^a^ antibodies rank among the most clinically significant alloantibodies, accounting for 20–30% of hemolytic transfusion reactions in recent UK Serious Hazards of Transfusion (SHOT) reports. These antibodies exhibit unique characteristics including complement-dependent hemolysis, dose-dependent serological reactivity, and rapid anamnestic responses, which collectively contribute to their high underdetection rates in routine screening [8,9]. While pregnancy and transfusion remain primary sensitization routes, the stealth nature of anti-Jk^a^ antibodies poses substantial risks-even subthreshold antibody levels undetectable by standard serology can trigger life-threatening intravascular hemolysis upon re-exposure to Jk(a+) erythrocytes [10,11]. This persistent diagnostic gap underpins the AABB (Association for the Advancement of Blood & Biotherapies)Guidelines mandating Jk(a-) blood for alloimmunized recipients regardless of current antibody detection status [12].

A particularly clinicopathological challenge arises from the weak Jk^a^ (Jk(a + ^w^)) phenotype, characterized by substantially reduced antigen expression that evades conventional serotyping [13]. First identified in 2011, this variant demonstrates ambiguous reactivity with anti-Jk^a^ reagents in standard tests, requiring protease treatment to reveal definitive positivity, thereby leading to frequent misclassification as Jk(a-) [14,15]. Multicenter studies reveal alarming rates of serological misidentification: 13.3% (4/30) in Taiwanese presumed Jk(a-b-) cohorts and 7.3% (20/275) in the Shenzhen blood donor population [13,16]. Clinically, transfusion of misclassified Jk(a + ^w^) units carries dual risks-de novo alloimmunization in naïve recipients and potentially fatal anamnestic reactions in pre-sensitized individuals. These findings collectively underscore the critical limitations of current serological paradigms and highlight the imperative for molecular diagnostic tests grounded in mechanistic understanding.

Molecular investigations into Jk(a + ^w^) pathogenesis remain controversial. Initial studies linked the phenotype to the JK*01W.01 allele (*c.130G > A*, p.Glu44Lys), proposing that *c.130G > A* might be a main cause to reduce Jk^a^ antigen density [14]. However, a striking paradox emerges from population studies. Despite 45.7% allelic frequency in Han Chinese versus 4.2% in Swedes, the actual Jk(a + ^w^) phenotype remains exceptionally rare in both populations. The results of the study imply an incomplete penetrance or epigenetic modifier effects [14,17]. Subsequent identification of the Asian-specific JK*01W.06 haplotype (*c.130G > A* coupled with *c.588A > G*) failed to resolve this enigma, as coding-sequence analyses demonstrated no causal relationship with antigen attenuation [18,19].

Emerging evidence implicates post-transcriptional regulation as a potential modulator, with RNA splicing variations influencing antigen presentation in multiple blood group systems including RHD [20], MNS [4], P1Pk [21] and Vel [22]. Intriguingly, recent work identified promoter polymorphisms in JK*01W.06 haplotypes associated with Jk(a + ^w^) phenotype [16], yet the structural diversity of *SLC14A1* transcripts and their functional impact on UT-B protein expression remain uncharacterized.

To address these knowledge gaps, we examined the structures of *SLC14A1* transcripts using PacBio long-read sequencing in Chinese blood donors, aiming to elucidate the mechanism underlying Jk(a + ^w^) presentation.

## Materials and methods

### Blood donor samples

The study was conducted during the period of 01/08/2022 to 31/05/2025. 18 volunteer Chinese blood donors, aged 25−55 in Shenzhen Blood Center, were recruited in this study (Fig 1). Fresh blood samples were collected after obtaining written informed consent, and the use of the samples was approved by Research Ethics Committee of Shenzhen Blood Center (Approval No: SZBCMEC-2022–013. Valid from July 16, 2022 to July 15, 2025).

**Fig 1:**
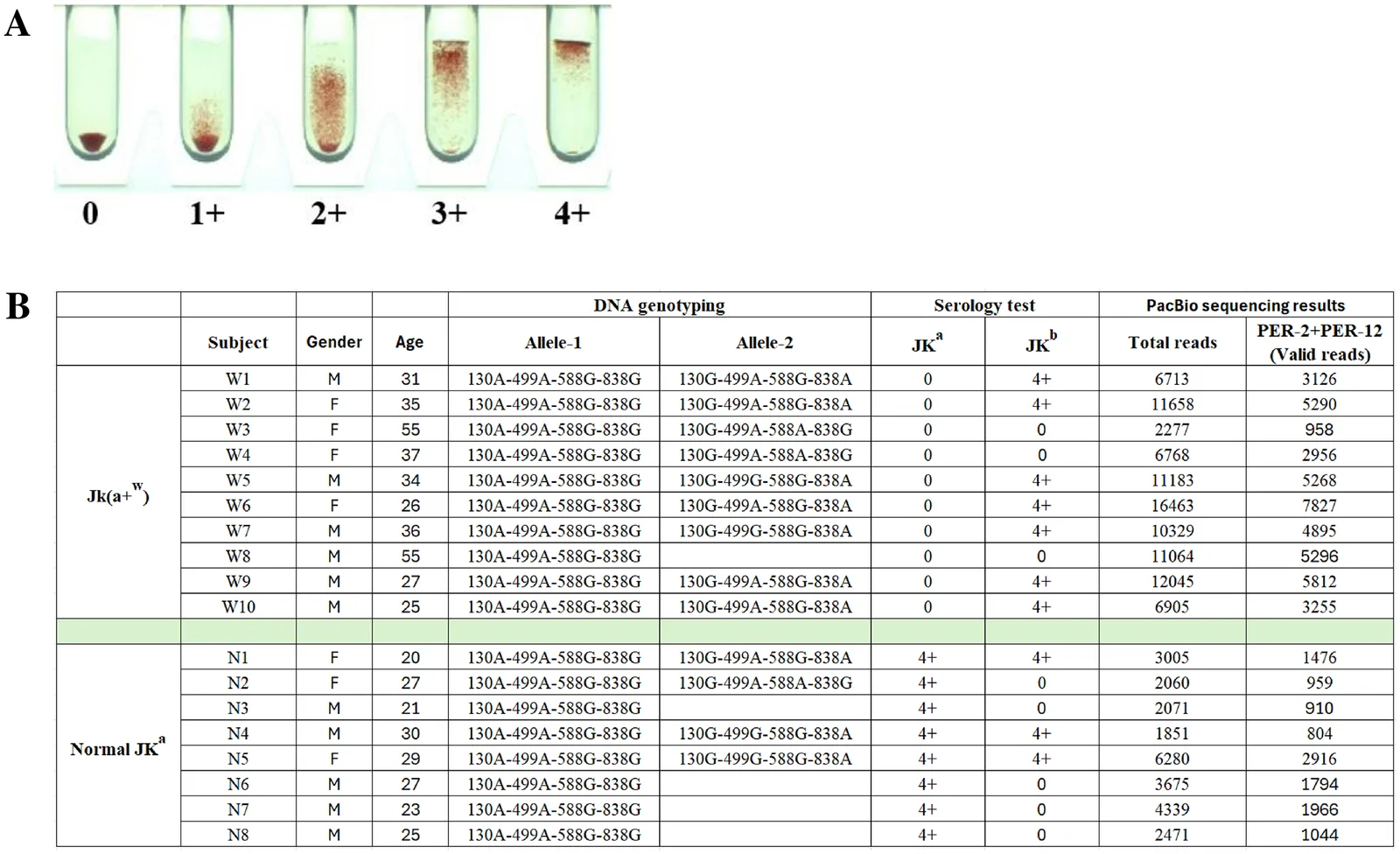
Clinical information of the research subjects. (A) Representative JK blood typing results using the gel column technology. (B) Clinical information of research subjects and the summary of PacBio sequencing reads.

Among the research subjects, 8 donors (N1-N8) were determined as normal Jk^a^ antigen presentation and 10 donors (W1-W10) showed Jk(a + ^w^) phenotype in serological tests with anti-Jk^a^ antibody (Fig 1). Each donor carried at least one JK*A allele (Fig 1B and S1 Fig) and the integrity of *SLC14A1* gene was confirmed via PacBio long-read sequencing with genomic DNA. In addition, haplotype blocks of four single nucleotide variants (SNVs) at nucleotide positions *130G/A*, *499A/G*, *588A/G* and *838G/A* in the *SLC14A1* ORF were also determined to differentiate two alleles of each subject (Fig 1B and S1 Fig).

Jk^a^ and Jk^b^ serological tests were performed using the monoclonal antibody products of CE-Immundiagnostika GmbH (Germany): Pelikloon anti-Jk^a^ (IgM) monoclonal from clone MS15, Pelikloon anti-Jk^b^ (IgM) monoclonal from clone MS8.

### RNA extraction and reverse transcription (RT)

In a preliminary experiment we examine the expression of blood type genes, *SLC14A1*, *GYPA* and *ABO*, in different types of blood cells after Ficoll-Hypaque (Merk/Sigma-Aldrich) separation. The mRNA of each gene was easily detected in red blood cells (RBC layer) due to the existence of reticulocytes, whereas the expression of *GYPA* and *ABO* genes could be hardly detected in nucleated blood cells (PBMC layer) (S2 Fig, S4 File). Hence in this study, total RNA extracted from RBC, instead of nucleated blood cells (PBMC) as reported previously [4], was used to amplify *SLC14A1* ORF fragments for sequencing analysis.Therefore, fresh blood samples were separated using Ficoll-Hypaque (Merk/Sigma-Aldrich) and anucleate red blood cells (RBC) were harvested. Total RNA was extracted from RBC with miRNeasy Serum/Plasma Kit (Qiagen) according to the manufacturer’s protocol.

500ng total RNA was used in a 20μl reverse transcription (RT) reaction to synthesize full length cDNA with SuperScript™ III reverse transcriptase (Thermo/Invitrogen).

To synthesize cDNA from synthesized RNA produced via *in vitro* transcription (IGE, Guangzhou, China), 200ng RNA was used in 20ul reverse transcription reaction with SuperScript™ III reverse transcriptase (Thermo/Invitrogen).

### PCR amplification and sequencing data analysis

*SLC14A1* ORF containing fragments were amplified via a two-round nested polymerase chain reaction (PCR) with EmeraldAmp® PCR Master Mix (Takara). In the first round PCR, 1μl of cDNA was used in a 25-μl reaction. The primers used for *SLC14A1* pre-amplification were forward 5’-ACTGGGCATCTCCTGCTAC-3’ and reverse 5’-ACATTCCAGTCTTAGTGCCA-3’. The PCR product was then used as the template and subjected to a second-round PCR with forward primer 5’-CAAAAACTACCTAAAATAAAGA-3’ and reverse primer 5’-GCTCAAGGATATTCCTGGAG-3’. The final PCR products were purified with PureLink PCR Purification Kit (Thermo/Invitrogen) and subjected to PacBio long-read sequencing performed by Haorui Genomics (Xian, China).

Sequencing data was then aligned with *SLC14A1* gene reference sequence extracted from Chr 18 (NCBI #NC_000018.10) (Fig 2A) and mRNA reference sequence (NM_015865.7) to determine exon composition of each transcript.

**Fig 2:**
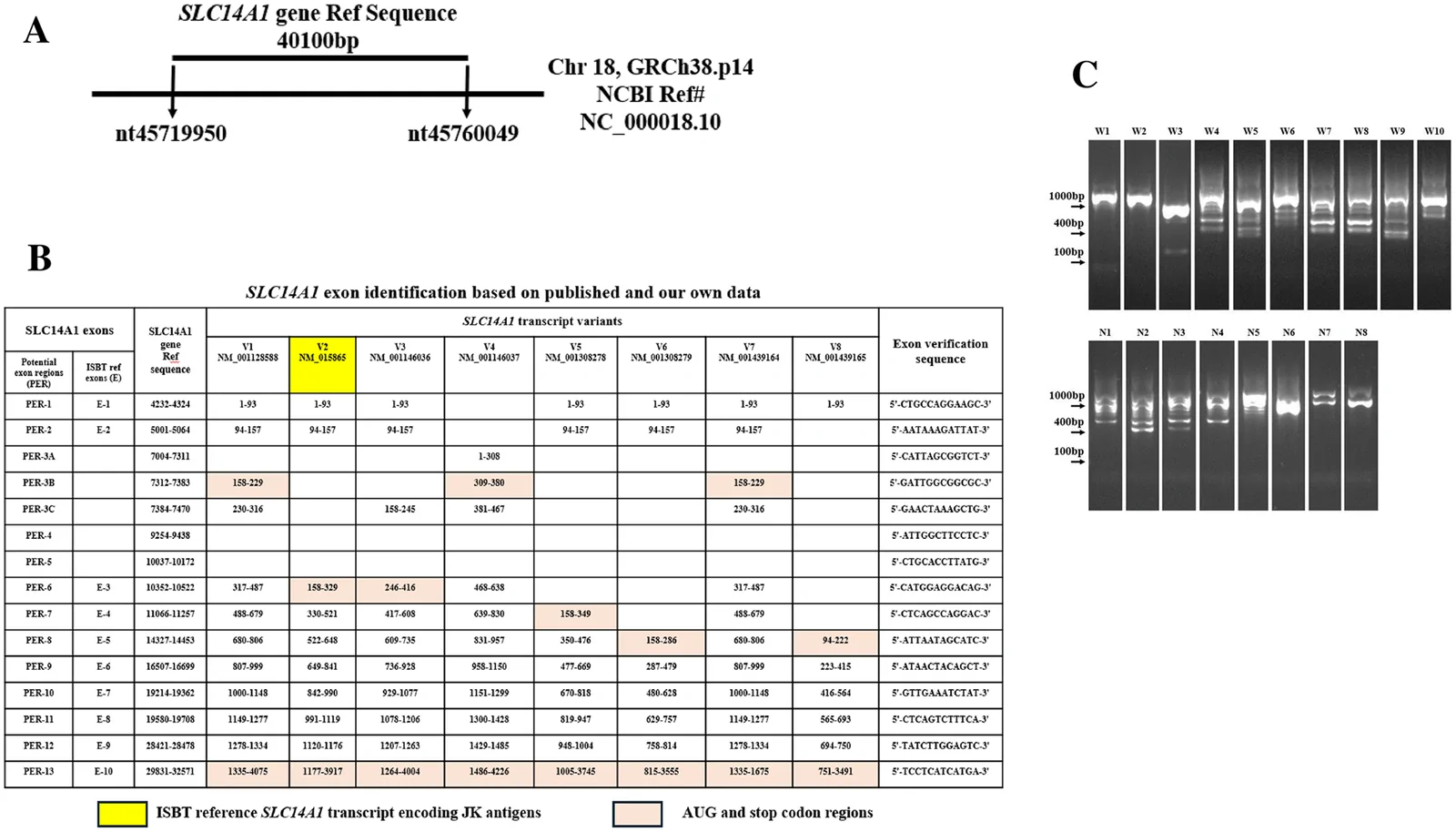
Summary of potential exon regions (PERs) of *SLC14A1* and fragment amplification for *SLC14A1* mRNA sequencing. (A) Schematic diagram depicting the location of reference sequence of *SLC14A1* gene in chromosome 18. (B) Summary of potential exon regions (PER) identified in *SLC14A1* gene. (C) RT-PCR amplification of the *SLC14A1* ORF fragments from the research subjects.

To examine the technical artifacts due to reverse transcription and PCR processes [23,24], two kinds of DNA templates were used to amplify *SLC14A1* fragments *via* PCR for PacBio sequencing analysis.

In the first experiment, *SLC14A1* fragments were directly amplified from sequence-confirmed plasmids.

In the second experiment, RNA was produced using T7 *in vitro* transcription system and subjected to reverse transcritption. Afterwards cDNA was used to amplify *SLC14A1* fragments for PacBio sequencing analysis. In the RNA template there were two gene cassettes containing tandem connected PER-6 and PER-7. The cassettes can be distinguished based on their haplotype styles of 130G/A-222C/A-326T/C of *SLC14A1* ORF, one possessed 130G-222C-326T and the other possessed 130A-222A-326C (S3 Fig).

### Statistics

Statistical analysis was performed using SPSS. Significant difference was determined by two-tailed Student’s t test, two-way analysis of variance followed by the Student’s t test.

## Results

### Fragment amplification for *SLC14A1* mRNA sequencing

According to the International Society of Blood Transfusion (ISBT), the JK antigens (Jk^a^ and Jk^b^) are encoded by the *SLC14A1* transcript variant 2 which contains 10 exons (NM_015865.7). Currently several *SLC14A1* splice variants have been reported hence we aligned their sequences with reference *SLC14A1* gene reference sequence (Fig 2B) trying to summarize all the potential exon regions (PER) in the *SLC14A1* gene that have been identified at present. Altogether there are 15 PERs, including two regions, PER-4 and PER-5, which were found in our work (Fig 2B). According to the guideline issued by ISBT, *AUG* codon of the ORF locates in PER-6, stop *TGA* codon locates in PER-13 and JK antigen-carrying codon (*838 A/G*) locates in PER-11.

PER-1 and PER-2 existed in almost all transcript variants except variant-4, which was very seldom detected in blood cells in our preliminary RT-PCR test. From PER-8 onwards, all the variants shared the same exon composition at 3’-end of *SLC4A1* transcripts. Therefore, we designed forward primers in PER-2 and reverse primers in PER-13 to ensure best coverage in the amplification of *SLC14A1* ORF fragments from the research subjects.

In addition, a well-conserved sequence (exon verification sequence, Fig 2B) in each exon has been identified via alignment of various *SLC14A1* sequencing results, including the reported data and those obtained in our work. The short sequences were used to examine the existence of corresponding exons in PacBio reads.

Based on the above-mentioned experimental design, *SLC14A1* ORF fragments were successfully amplified from all the research subjects (Fig 2C, S4 File). The collected PacBio sequencing reads (S1 and S2 Files) were first filtered with PER-2 and PER-12 verification sequences (Fig 2B) to obtain valid reads containing *838G/A* sites (PER-2+PER-12 reads) for *SLC14A1* mRNA structure analysis (Fig 1B). As expected about half of the total reads were extracted as valid reads because double strand PCR products were input for PacBio sequencing.

Afterwards each potential exon region (PER, Fig 2B) was split into ~20 bp pieces and the short sequences were used to preliminarily filter the *SLC14A1* transcript reads of the research subjects to examine the exon composition and nucleotide variation in each piece of transcript (S1 Table).

### Changes of exon combination in *SLC14A1* transcripts

The exons summarized in Fig 2B except PER-3A were all detected in the PacBio sequencing reads (Table 1). Among them, PER-4 and PER-5 were newly identified in our work. PER-4 inclusions were observed in 19.16% of the valid reads of subject N8 and led to frameshift mutations compared to the ORF of reported *SLC14A1* transcript variant 1 (NM_001128588.4). PER-5 inclusions were observed in 12.17% of the valid reads of subject W10 and introduced a novel *AUG* start codon and frameshift mutations compared to the ORF of reported *SLC14A1* transcript variant 2, the reference JK antigen-encoding mRNA proposed by ISBT (NM_015865.7).

**Table 1:** Summary of the exon compositions of the *SLC14A1* ORF in the research subjects.

		Total reads	Valid reads	PRE-2	PER-3A	PER-3B	PER-3C	PER-4	PER-5	PER-6	PER-7	PER-8	PER-9	PER-10	PER-11	PER-12
**Jk(a +** ^**w**^)	**W1**	**6713**	**3126**	**3126**	**0**	**0**	**0**	**0**	**0**	**3055**	**3039**	**3055**	**3057**	**3049**	**3013**	**3126**
	**W2**	**11658**	**5290**	**5290**	**0**	**0**	**0**	**0**	**0**	**5074**	**5090**	**5103**	**5113**	**4999**	**5064**	**5290**
	**W3**	**2277**	**958**	**958**	**0**	**0**	**0**	**0**	**0**	**937**	**938**	**933**	**3**	**3**	**3**	**958**
	**W4**	**6768**	**2956**	**2956**	**0**	**0**	**0**	**0**	**0**	**2620**	**2590**	**2893**	**2830**	**2862**	**2848**	**2956**
	**W5**	**11183**	**5268**	**5268**	**0**	**0**	**0**	**50**	**23**	**3324**	**3316**	**5081**	**5102**	**5071**	**5081**	**5268**
	**W6**	**16463**	**7827**	**7827**	**0**	**87**	**89**	**146**	**0**	**6860**	**7044**	**7622**	**7601**	**7522**	**7618**	**7827**
	**W7**	**10329**	**4895**	**4895**	**0**	**2**	**2**	**0**	**0**	**3579**	**3543**	**4715**	**4646**	**4583**	**4552**	**4895**
	**W8**	**11064**	**5296**	**5296**	**0**	**1395**	**1394**	**0**	**0**	**4540**	**4610**	**5194**	**5078**	**4935**	**5034**	**5296**
	**W9**	**12045**	**5812**	**5812**	**0**	**255**	**451**	**0**	**0**	**5159**	**5293**	**5682**	**5557**	**5427**	**5415**	**5812**
	**W10**	**6905**	**3255**	**3255**	**0**	**0**	**0**	**0**	**396**	**2219**	**2608**	**3181**	**3196**	**3123**	**3167**	**3255**
**Normal Jk** ^**a**^	**N1**	**3005**	**1476**	**1476**	**0**	**23**	**24**	**3**	**12**	**608**	**712**	**1386**	**1075**	**1172**	**1160**	**1476**
	**N2**	**2060**	**959**	**959**	**0**	**8**	**7**	**1**	**0**	**226**	**242**	**782**	**532**	**593**	**613**	**959**
	**N3**	**2071**	**910**	**910**	**0**	**21**	**33**	**5**	**10**	**302**	**440**	**855**	**795**	**746**	**750**	**910**
	**N4**	**1851**	**804**	**804**	**0**	**0**	**0**	**0**	**0**	**351**	**394**	**772**	**595**	**588**	**600**	**804**
	**N5**	**6280**	**2916**	**2916**	**0**	**365**	**421**	**148**	**61**	**2104**	**2177**	**2826**	**2714**	**2713**	**2719**	**2916**
	**N6**	**3675**	**1794**	**1794**	**0**	**28**	**31**	**44**	**0**	**379**	**871**	**1734**	**1237**	**1449**	**1480**	**1794**
	**N7**	**4339**	**1966**	**1966**	**0**	**0**	**0**	**0**	**0**	**1081**	**1042**	**1876**	**1900**	**1882**	**1896**	**1967**
	**N8**	**2471**	**1044**	**1044**	**0**	**408**	**401**	**200**	**0**	**408**	**407**	**1006**	**1010**	**1003**	**990**	**1044**

Sequences used in exon screening were described in Fig 2B. ORF: Open reading frame. PER: Potential exon region.

In the *SLC14A1* mRNA of each research subject, different kinds of exon combinations were identified based on the analysis of the frequency of each exon in the transcripts, proposing a mixture of different splicing variants in the samples. For example, in total 4895 valid reads of W7, PER-6 was detected in 3579 reads whereas PER-8 appeared in 4715 reads, indicating at least two kinds of splicing variants existed, transcripts with PER-6 and transcripts without PER-6 (Table 1).

In *SLC14A1* mRNA of the subject W3, PER-9 to PER-11 were skipped completely (Table 1) although the correspondence regions could be detected in genomic DNA (S1 Fig). To exclude the possibility that the unusual splicing was caused by genetic mutations, flanking regions adjacent to the potential exon regions were sequenced and no mutations were detected at the exon-intron junctions (S4 Fig). Since JK antigen site (*838 G/A*) locates in PER-11, hence the exon skipping may lead to total loss of the JK antigen site. Therefore, the weak Jk^a^ antigenic phenotype of subject W3 may be directly caused by PER-11 skipping.

Interestingly, PER-6 skipping and PER-7 skipping were much more frequently detected in normal Jk^a^ subjects compared to that in the Jk(a + ^w^) subjects (Fig 3). In normal subjects, only 40% of the valid reads contained PER-6, whereas the exon was detected in more than 80% of the valid reads of Jk(a + ^w^) subjects (Fig 3A). Similar results were obtained when comparing *Jk*^*a*^allele-encoded transcripts extracted from the valid reads of normal Jk^a^ group and Jk(a + ^w^) group (Fig 3B), indicating the alternative splicing was not affected by JK antigen-carrying site. PER-6 skipping and PER-7 skipping may result in the synthesis of the truncated SLC14A1 proteins (Fig 3C).

**Fig 3:**
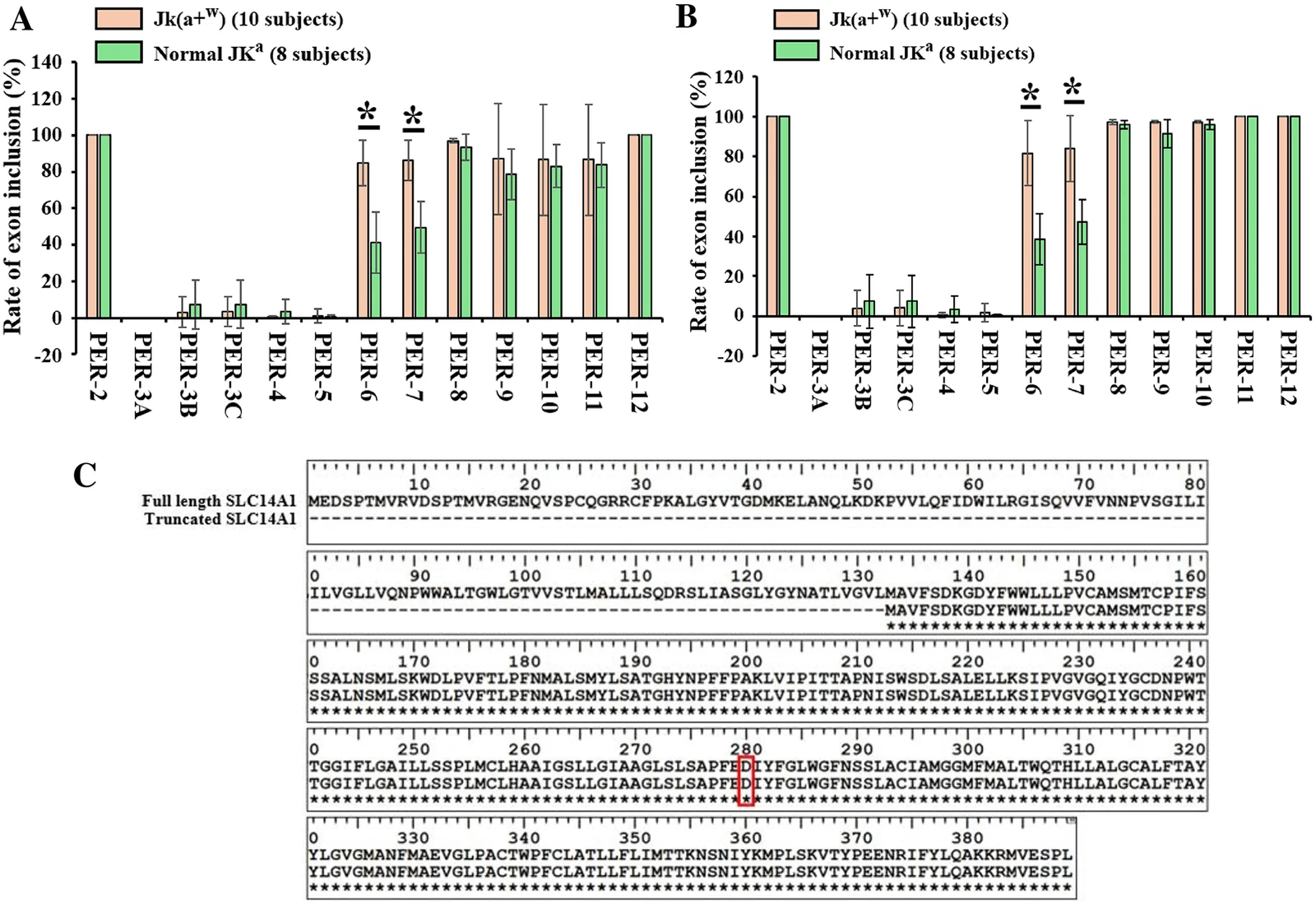
PER-6 skipping and PER-7 skipping were frequently observed in the *SLC14A1* transcripts of normal Jk^a^ subjects. (A) Inclusion rates of PER-6 and PER-7 were significantly higher in *SLC14A1* transcripts of Jk(a + ^w^) subjects. * p < 0.05. (B) Inclusion rates of PER-6 and PER-7 were significantly higher in *SLC14A1* transcripts encoded by JK*A alleles of Jk(a + ^w^) subjects. * p < 0.05. (C) Alignment of the full length SLC14A1 protein encoded by *SLC14A1* variant 2 mRNA and the truncated isoform encoded by *SLC14A1* variant 6 mRNA. Red frame indicates the amino acid carrying JK antigenicity.

### Transcription error-induced indels in the *SLC14A1* transcripts of the Jk(a + ^w^) subjects

Preliminary screening of the PacBio sequencing data also revealed the existence of nucleotide insertions and deletions (indels) in PER-6, PER-9 and PER-11 of the *SLC14A1* transcripts (S1 Table). As the representatives, the “G” deletion in mRNA of W2 subject and the “A” insertion in mRNA of N7 subject were clearly verified in the Sanger sequencing results. Further examinations showed that the indels were only detected in mRNA but not in genomic DNA, indicating the indel mutations were caused by transcription errors (Fig 4A).

**Fig 4:**
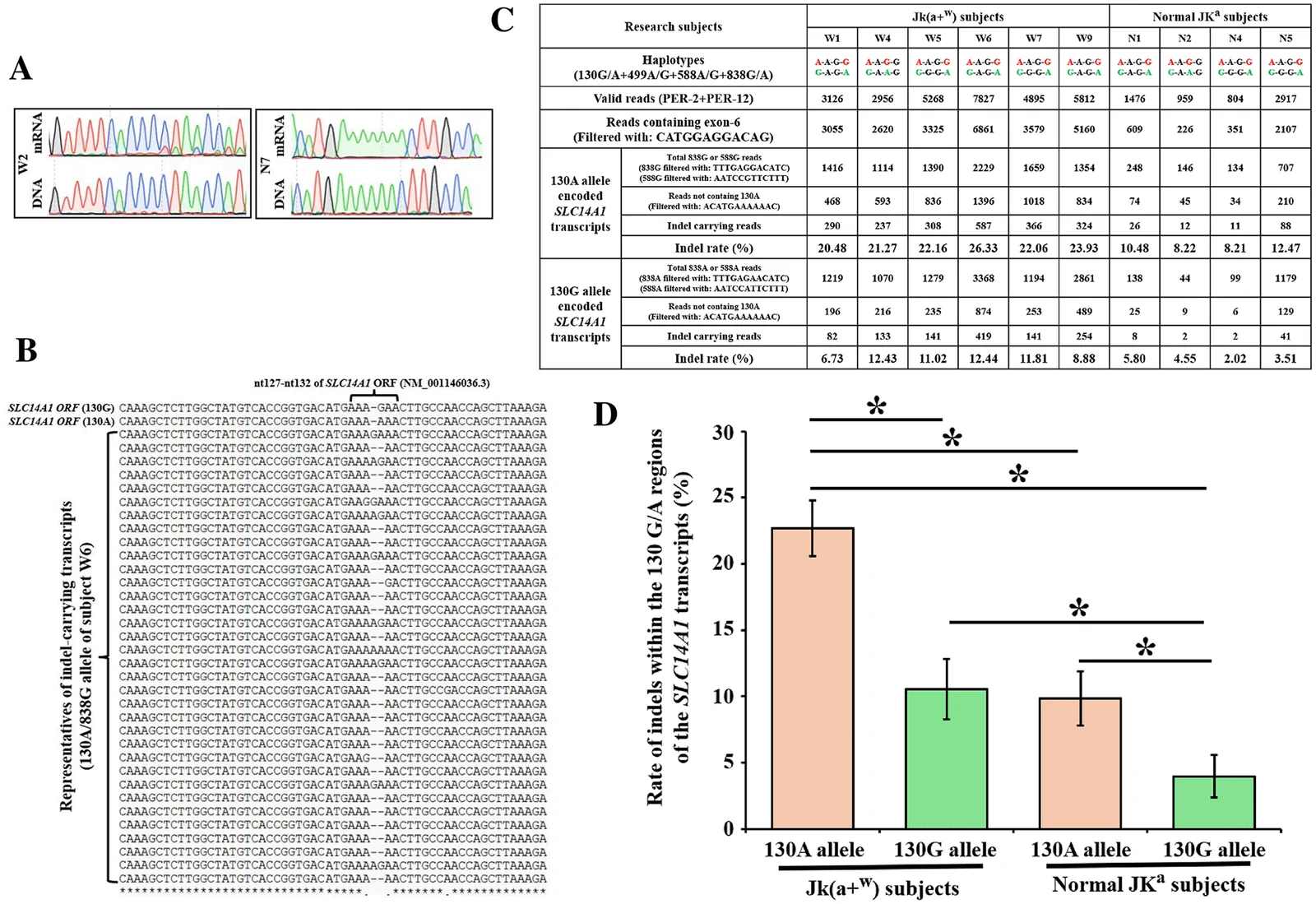
Indels were frequently detected in the *SLC14A1* transcripts of the Jk(a + ^w^) subjects. (A) Representative sequencing chromatograms showing the indels that appeared in mRNA but not in the corresponding regions in genomic DNA. The minor peaks in the Sanger chromatogram of W3 mRNA represent sequences of transcripts with “C” deletion. (B) Representative alignment of the *SLC14A1* of subject W6 showing the indels that were induced during transcription at the position of the SNV *130 G/A*. (C)&(D) *130A* was more susceptible for transcriptional indel induction and the indel rates at the position *130A* were significantly higher in Jk(a + ^w^) subjects. * p < 0.05.

In addition, higher mismatching rates were observed within the regions around the position of the single nucleotide variant (SNV) *130G/A* in the transcripts of Jk(a + ^w^) individuals (S1 Table). Alignment of those mismatching sequences revealed frequent indel mutations at this nucleotide position (Fig 4B).

To further examine whether the transcriptional indel mutations around the *130G/A* position may contribute to the discrepancy of the JK antigen presentation, Jk(a + ^w^) and normal Jk^a^ subjects with the following features were selected. First, the subjects had heterozygous genotypes at position *130G/A*. Hence subjects W8, N3, N6, N7 and N8 were excluded. Second, there were no alternative *AUG*-carrying exons that could cause frameshifts in *SLC14A1* ORF. Hence subject W10 was excluded. Third, there were no other obvious defects that could lead to reduced JK antigen expression. Hence subjects W2 and W3 were excluded. In the end 6 Jk(a + ^w^) subjects and 4 normal Jk^a^ subjects were selected for this analysis.

Next, sequential filtration was performed to examine the rates of indel generation in the regions around the *130G/A* position in the *SLC14A1* transcripts of each subject. For example, the indel rates of subject W6 were evaluated via the following stepwise analysis method (Fig 4C): First, 6861 pieces of PER-6 containing valid reads were filtered out with verification sequences of PER-2, PER-12 and PER-6. Second, 2229 transcripts carrying *130A* were obtained via filtration with *838G*-containing sequence based on the haplotype patterns listed in Fig 1B. Third, 1396 reads that did not fully match the reference sequence in the region around the *130A* position were screened out. Fourth, the 1396 pieces of sequence were aligned and 587 pieces that contained indel mutations (Fig 4B) were discovered. In the end, the indel rate within the *130A* region of subject W6 was calculated by dividing 587 indel-containing reads by 2229 *130A* allele derived reads, that was, 26.33%. The indel rate within the *130G* region of the transcripts derived from the other allele of subject W6 was obtained in the same way, that was, 12.44%.

For some subjects, such as W4 and N2, haplotype analysis showed that *130A* was linked with *588G* in one allele and *130G* was linked with *588A* in the other allele. Hence *588G* containing sequence was used to screen *130A*-allele derived transcripts and *588A* containing sequence was used to screen *130G*-allele derived transcripts (Fig 4C). The 12-bp sequences used for sequential filtration were listed in Fig 4C.

Indel rates within the *130A*-containing regions were significantly higher than those within the *130G*-containing regions in all the subjects, no matter whether they were Jk(a + ^w^) subjects or normal Jk^a^ subjects (Figs 4C and 4D). In addition, indel mutations occurred much more frequently within the regions around the *130G/A* position compared to the adjacent regions, which was consistent with the observation in the preliminary screening (S1 Table). The results indicated that in the *SLC14A1* gene, the sequence containing nucleotide *130A* was more prone to induce transcription errors than that containing nucleotide *130G*.

Interestingly, indel rates within the *130A*-containing regions of the Jk(a + ^w^) subjects were significantly higher than that of the normal Jk^a^ subjects. Similarly, indel rates within the 130G-containing regions also increased in the Jk(a + ^w^) subjects compared to the normal Jk^a^ subjects (Fig 4D). The results strongly suggested that the transcription fidelity was significantly lower in the Jk(a + ^w^) subjects than the normal Jk^a^ subjects.

To exclude the possibility that the above mentioned indels were just RT-PCR artifacts rather than true transcription errors [23,24], plasmid templates and synthesized RNA templates were employed to assess the indel rates generated in reverse transcription and PCR processes (S3 Fig, S1 Table, S4 File). RNA-derived PCR products showed higher mismatching rates and indel rates compared with the PCR products directly amplified from plasmid template, indicating reverse transcription may really induce mutation artifacts. However the indel rates in the 6-nucleotide region were much lower than that observed in research subjects, indicating RT-PCR artifacts can be ignored when analysing the transcription errors in this study (Fig 4, S3 Fig).

## Discussion

Currently the molecular mechanisms underlying the Jk(a + ^w^) phenotype are still not clear and genomic DNA-based studies have shown limited and controversial results. Compared to genome DNA, RNA is significantly more dynamic in the senses of expression levels and molecule structures, which are highly susceptible to environmental and cellular changes [25]. Consistently, it has been found that alternative RNA splicing may directly shape cell surface antigenicity in not only cancer cells [26], but also red blood cells [27,28]. Therefore, the examination of *SLC14A1* mRNA molecules may help to elucidate the bewildering phenomena observed in the JK antigen study, such as heterogeneous and evanescent properties of Kidd antibodies [29,30]. Hence in this study we characterized the *SLC14A1* mRNA trying to investigate whether there were some structural changes of *SLC14A1* mRNA that might contribute to the variation of Jk^a^ antigen presentation.

It is very interesting to find that a significant portion of the transcripts in the normal Jk^a^ subjects possess the exon combination pattern of *SLC14A1* transcript variant 6 but not the variant 2 which is assigned as the reference mRNA encoding the Jk antigen-carrying SLC14A1 protein by ISBT (Table 1, Fig 3). In transcript variant 6 PER-6 and PER-7 are skipped, hence a new *AUG* start codon is introduced and leads to the translation of a truncated isoform of SLC14A1 protein [31]. Further work needs to be done to verify the efficiency of the novel *AUG* start codon in initiating the translation as there are two upstream *AUG* codons (*uAUG*) in the 5’-untranslated region (5’-UTR) [32], similar to the mRNA of the *PRPH* gene [33]. In addition, it is worth comparing the Jk^a^ antigenicity carried by different SLC14A1 protein isoforms produced by alternative splicing to elucidate the mechanism underlying the variation of Jk^a^ antigen presentation.

The loss of JK antigen-carrying PER-11 has been obviously observed, *i.e.*, in more than 10% of the valid reads, in not only the Jk(a + ^w^) subjects W3, but also a number of normal Jk^a^ subjects, such as N1, N2 and N6 (Table 1). However, only subject W3 in which PER-11 was skipped in all the transcripts showed Jk(a + ^w^) phenotype (Fig 1). It will be interesting to examine the threshold proportion of PER-11 deletion that may lead to Jk(a + ^w^) phenotype to explain the dosage effect encountered in serological test [34].

Conspicuous exon insertions that may cause frameshift mutations were also observed, such as PER-5 inclusion in W10 and PER-4 inclusion in N8. Interestingly, in the normal Jk^a^ subject N8, PER-4 were always found on PER-6 containing transcripts whereas most transcripts of this subject possessed the exon combination pattern of *SLC14A1* transcript variant 6 in which PER-6 and PER-7 were skipped and *AUG* start codon located in PER-8. The results strongly support the speculation that the transcript variant 6-encoded SLC14A1 protein isoform contributes to the creation of the Jk^a^ presentation.

Transcription errors can disrupt RNA structures [35] and have been reported to play significant roles in age-related diseases [36] and cancers [37]. In this study we have also observed transcription error-related changes in the *SLC14A1* transcripts, including nucleotide substitution mutations and indel mutations. Interestingly, we found that transcription errors occurred in a sequence-dependent pattern but not a random pattern as reported previously [35]. We found that in the 6-nucleotide region around SNV *130G/A* in the *SLC14A1* gene, *130A* allele-encoded transcripts showed a higher indel rate compared to the transcripts encoded by *130G* allele (Fig 4), possibly due to the fact that sequences of consecutive identical nucleotides were more susceptible to transcription errors and transcription slippage efficiency was largely influenced by the upstream and downstream bases [38]. Indel rates at the position *130A* were significantly higher in the Jk(a + ^w^) subjects than in the normal Jk^a^ subjects, indicating there are some risk factors influencing the transcription fidelity in the Jk(a + ^w^) individuals. Therefore, *130A* may play roles in promoting Jk(a + ^w^) phenotype only when the transcription fidelity is reduced. Our results indicate that mRNA characterization may help to address some controversial issues observed in genomic DNA-based studies, *e.g.*, not all *130A* bearing individuals demonstrate Jk(a + ^w^) phenotype [14,17,39].

Interestingly, in the normal Jk^a^ subject N7, although “A” insertion at the position *130A* was observed in all the transcripts containing PER-6, about half of the transcripts possessed the exon combination of the variant 6 pattern (Table 1, S1 Table). Therefore, the individual N7 demonstrated normal Jk^a^ phenotype possibly due to the exon-6 and exon-7 skipping and the introduction of a novel *AUG* start codon in exon-8 (Fig 3C). It is another example to support the speculation that the transcript variant 6-encoded SLC14A1 protein isoform contribute to the creation of the Jk^a^ presentation. In addition, N7 is a homozygous *Jk*^*a*^ individual (Fig 1B), hence “double dose” effects might be another factor contributing to normal Jk^a^ antigen presentation in N7.

Besides the nucleotide position *130A*, some other transcription error-prone positions were also identified. But indel occurrences at those positions were not as common as that at the *130A* position. For example, “G” deletion at nucleotide position 50 of the *SLC14A1* ORF was only observed in subject W2, and *838G/A* deletion was only observed in several Jk(a + ^w^) subjects (S1 Table). In some Jk(a + ^w^) subjects, indels may occur at different positions and cumulatively contribute to the reduced Jk^a^ antigen presentation.

Therefore, transcription errors play important roles in the weak Jk^a^ presentation. Further study needs to be conducted to investigate the underlying mechanism of the transcription errors observed in the Jk(a + ^w^) individuals [40].

We have also found that the alternative splicing at the 5’ end of *SLC14A1* mRNA is more complex than it has been discovered so far. Therefore 5’ RACE needs to be performed to reveal other potential 5’ ends of *SLC14A1* transcripts. In addition, methods to examine the gene expression level of *SLC14A1* in erythrocytes, such as RT-qPCR analysis, should be established to better elucidate the mechanisms underlying weak Jk^a^ antigen presentation.

Maintenance of transcriptional fidelity depends on the stability of POL II complex composed of 12 subunits that may be dysregulated under certain conditions, such as cancers and other diseases [25]. For example, dysregulation of RPB9 gene has been shown to cause reduced transcription fidelity [37,41]. Similarly, RNA splicing is also highly dynamic, depending on the regulations of the spliceosome complex under various conditions [42]. Therefore, it is necessary to examine RNA status at certain time points when determining the blood group antigenicity in clinical practice. For example, in W3 subject JKa antigen-encoded PER11 was skipped in all transcripts (S1 Table). However, due to the confirmed integrity of SLC14A1 gene (S1 Fig, S4 Fig), it seems possible that Jk(a + w) phenotype may be changed under certain physiological conditions. Hence, mRNA characterization might be an invaluable supplement to serological phenotyping and DNA-based genotyping at the time of blood transfusion.

In this study, some strategic concerns were considered when performing experiments. First, RNA was extracted from erythrocyte layer instead of PBMC samples (S2 Fig). This is to improve the RNA source specificity for blood group antigen studies and avoid the problems that we have ever encountered in the previous work [4]. Second, *SLC14A1* transcripts were amplified via RT-PCR for sequencing instead of whole transcriptome sequencing. This is to guarantee enough target input for sequencing and the coverage of various target transcripts, especially when the gene expression level is relatively low. Third, sequential filtering and repeated alignment of mutation-carried sequence reads were performed instead of high-throughput bioinformatical analysis. This is to improve the resolution and accuracy of the detailed changes of target sequences. Moreover, the analysis method that we have established is more straightforward for bench-top researchers and they can easily add their findings and thoughts in the analysis process to obtain more detailed information. Actually, by doing this we have identified the indels in the 6-nucleotide region around 130G/A position (Fig 4). Therefore the methods in this work are surely helpful for those researchers who intend to do similar investigations.

In conclusion, our results have demonstrated that alternative splicing and transcription errors are important risk factors for weak Jk^a^ antigen presentation. *SLC14A1* transcript characterization might be an invaluable supplement to traditional serological phenotyping and DNA-based genotyping in KIDD blood grouping.

## Supporting information

S1 Fig Genotyping of single nucleotide variants (SNVs) of the human subjects in the study. 4 loci of the frequently mentioned SNVs in *SLC14A1* gene were amplified and subjected to Sanger sequencing. The results confirmed the genotyping results with PacBio sequencing with genomic DNA that is described in “MATERIALS AND METHODS”.
(TIFF)

S2 Fig Examination of the expression of the blood group antigen-encoding genes in different blood cell types. PBMC and RBC cells were collected from Ficoll-Paque separation and RNA was extracted from the cells. RT-PCR was performed to amplify the fragments of various blood group antigen-encoding mRNA. (A) Schematic diagram of the separation of the components of a blood sample from Ficoll-Paque density gradient medium. (B) Cell counting and yield of RNA extraction. (C) RT-PCR results of various blood group antigens.
(TIFF)

S3 Fig Examination of RT-PCR artifacts using *in vitro* synthesized RNA. (A) Schematic diagram of the synthesized RNA template. (B) RNA was produced using T7 in vitro transcription system and purified for RT-PCR. (C) DNA Fragments amplified from synthesized RNA templates *via* RT-PCR. (D) Representative alignment of the PacBio reads showing the indels that were induced in RT-PCR at the position of the SNV 130 G/A of *SLC14A1* gene. (E) Summary of indel rates generated in RT-PCR with synthesized RNA templates.
(TIFF)

S4 Fig Examination of exon-intron junctions with genome DNA of Subject W3. Adjacent regions flanking *SLC14A1* potential exon regions (PER) were amplified by PCR from genome DNA and subjected to sanger sequencing.
(TIFF)

S1 Table Screening for exon inclusions and nucleotide mismatches in the *SLC14A1* transcripts of the research subjects. PER, potential exon region.
(XLSX)

S1 File Sequencing data of normal Jk^a^ subjects. (ZIP)

S2 File Sequencing data of weak Jk^a^ subjects. (ZIP)

S3 File Sequencing data of *SLC14A1* plasmids and *in vitro* synthesized RNA templates. (ZIP)

S4 File Underlying raw images for the gel results. (PDF)
