# Supplementary material for: Alterations of SLC14A1 mRNA structure are frequently detected in weak Jka presenting donors
Source: PLoS One. 2026 Oct 14;21(10):e0360437. doi: 10.1371/journal.pone.0360437 (PMC13667791; doi:10.1371/journal.pone.0360437)
Supplement: Supplementary file 1 [file pone.0360437.s009.pdf]

For Fig 2

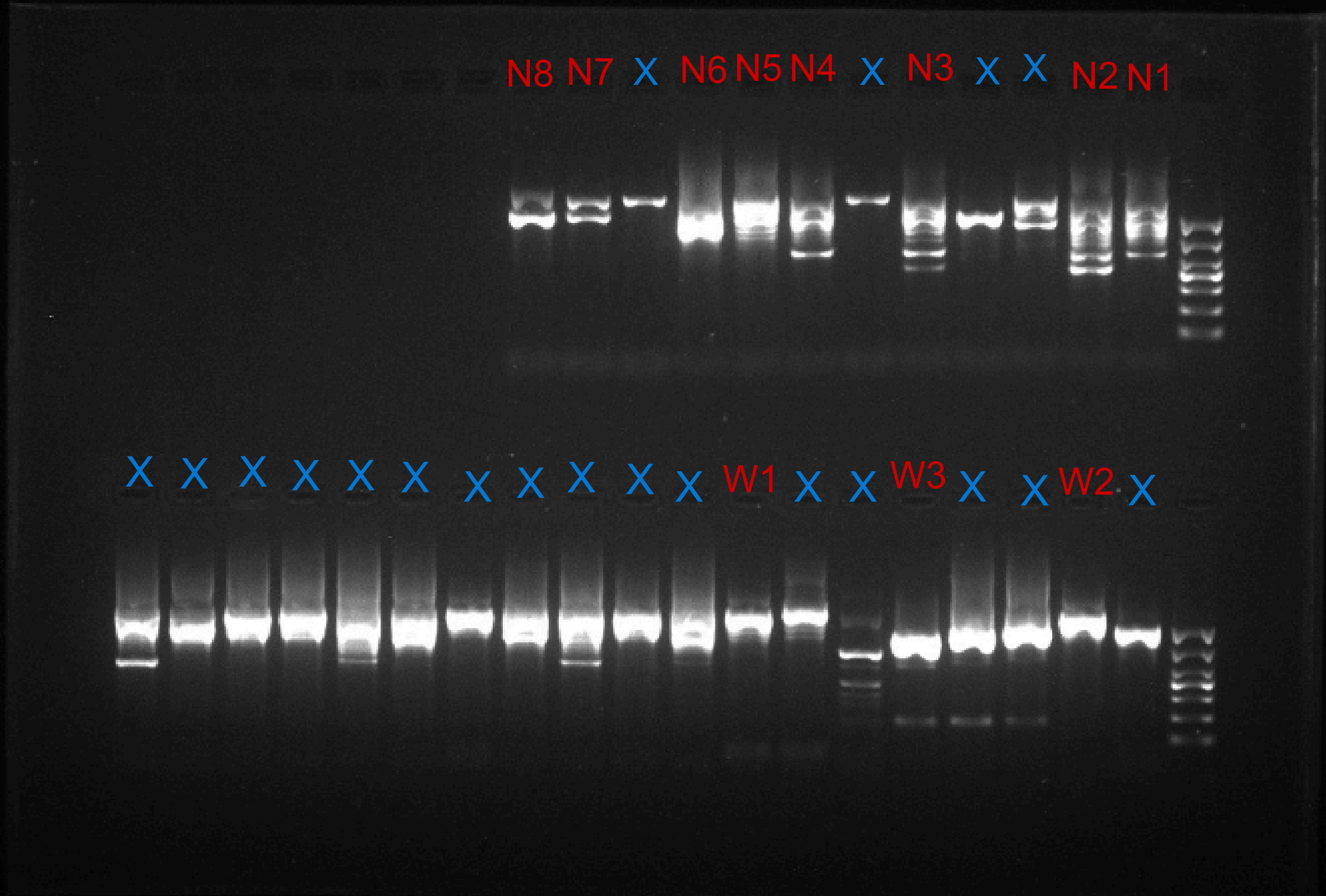

For Fig 2

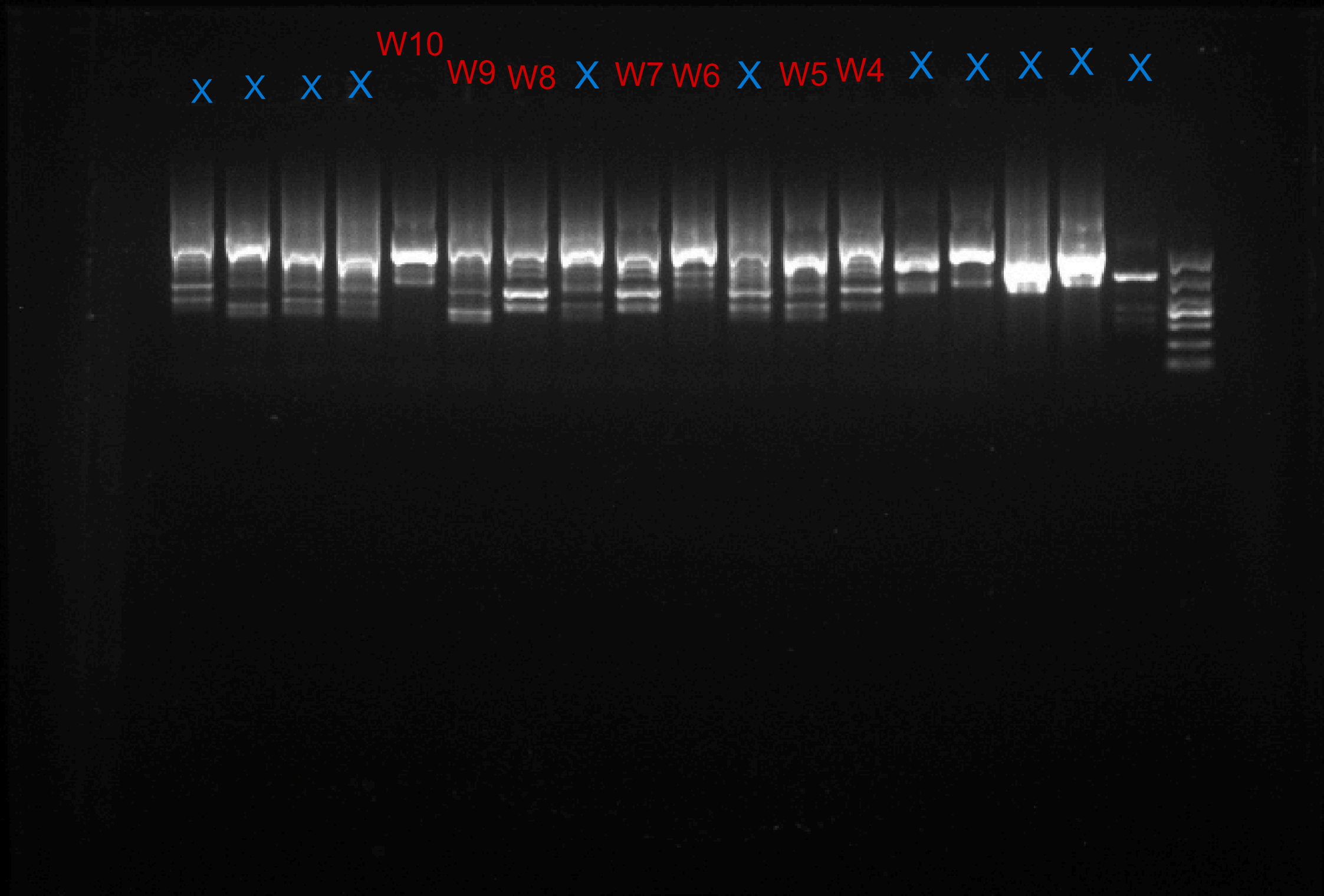

For S2 Fig

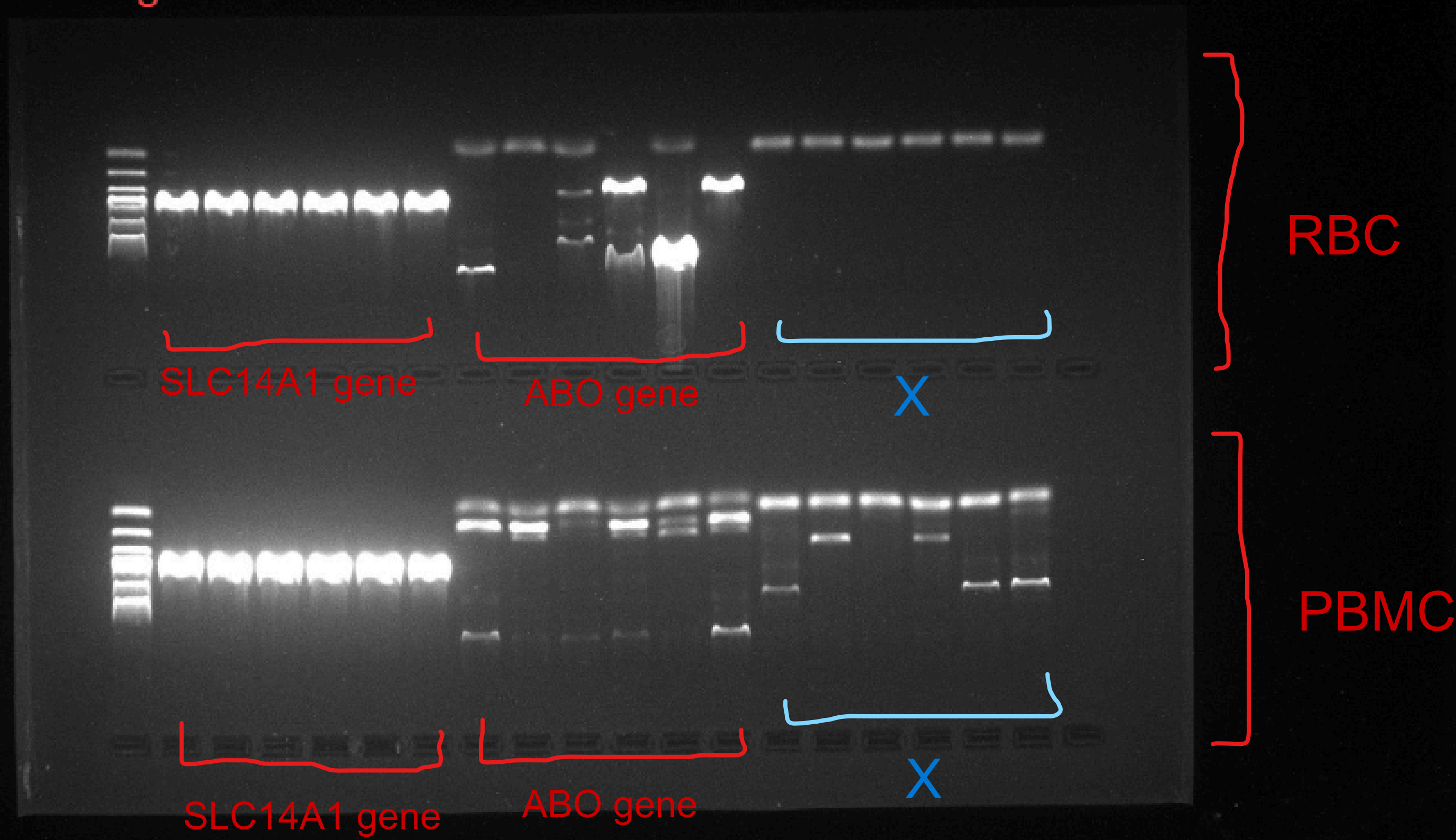

For S2 Fig

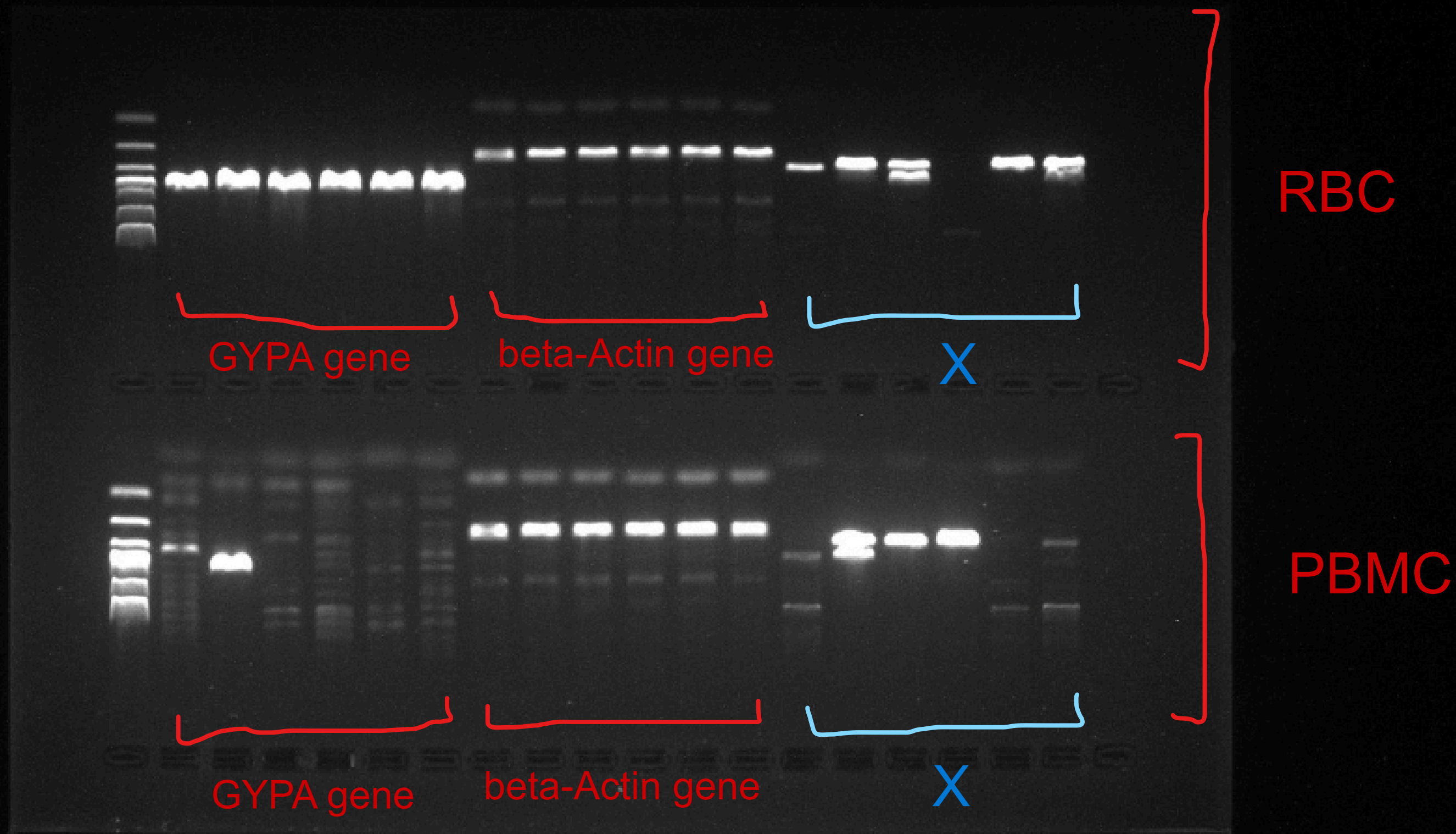

GYPA gene
beta-Actin gene
X
RBC
GYPA gene
beta-Actin gene
X
PBMC

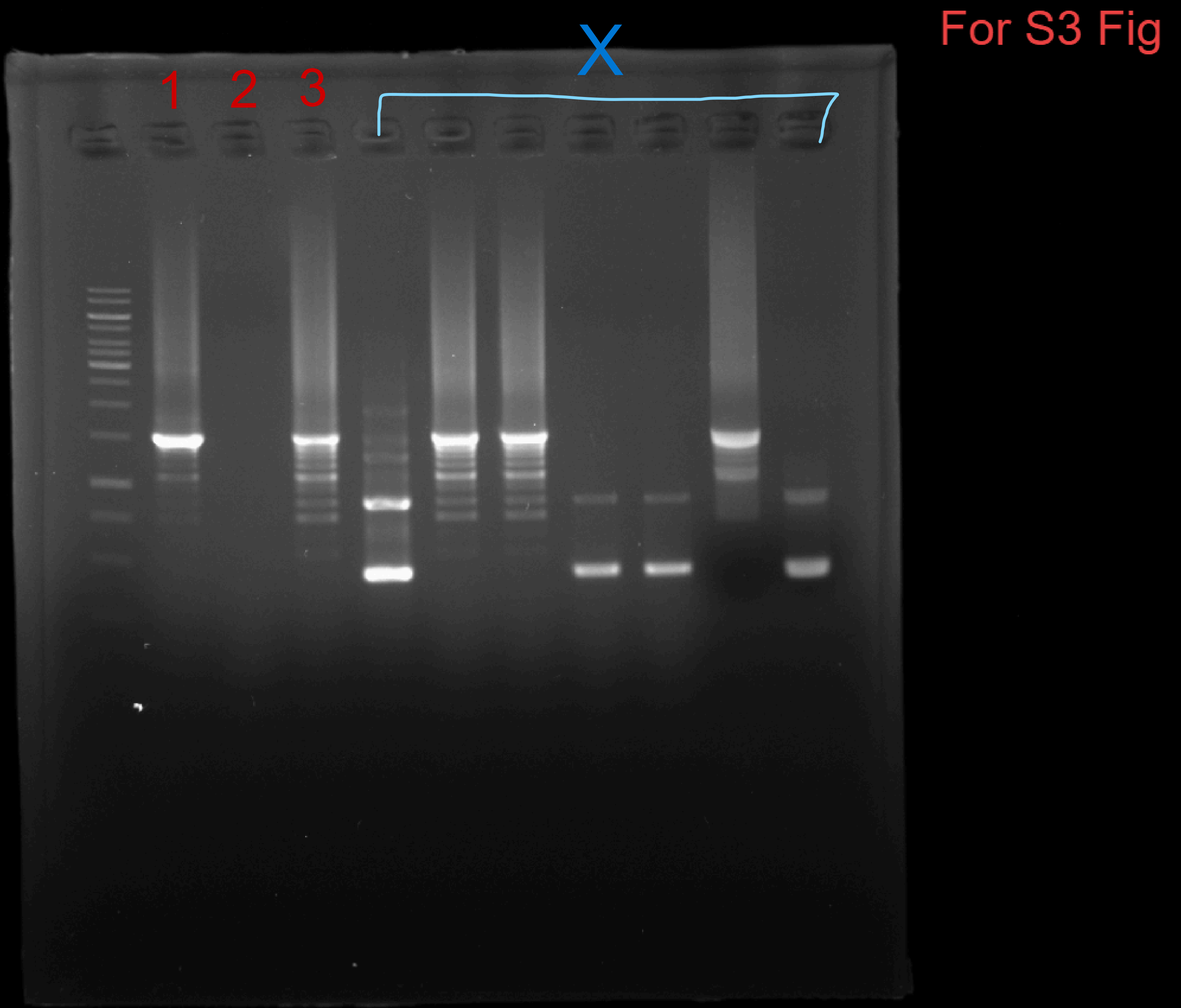
For S3 Fig
X
1
2
3
