# Supplementary material for: Alterations of SLC14A1 mRNA structure are frequently detected in weak Jka presenting donors
Source: PLoS One. 2026 Oct 14;21(10):e0360437. doi: 10.1371/journal.pone.0360437 (PMC13667791; doi:10.1371/journal.pone.0360437)
Supplement: Supplementary file 2 [file pone.0360437.s011.docx]

**Response to Reviewer Marks on the manuscript**

**1. Is the manuscript technically sound, and do the data support the conclusions?**

**Reviewer #1: No	Reviewer #2: Partly**

In this study, we conducted experiments based on following strategic concerns:

First, RNA was extracted from erythrocyte layer instead of PBMC samples. This is to improve the RNA source specificity and avoid the problems in subsequent RT-PCR amplification of blood group antigen gene transcripts that we have ever encountered in previous work (PMID: 35138639).

Second, SLC14A1 transcripts were amplified via RT-PCR for sequencing instead of whole transcriptome sequencing. This is to guarantee enough target input for sequencing and the coverage of various target transcripts, especially when the gene expression level is relatively low.

Third, sequential filtering and repeated alignment of mutation-carried sequence reads were performed instead of high-throughput bioinformatical analysis. This is to improve the resolution and accuracy of the detailed changes of target sequences. Moreover, the analysis method that we have established is more understandable and easy-going for bench-top researchers and they can easily add their findings and thoughts in the analysis process to obtain more detailed information.

Therefore, we think the conclusions in our manuscript can be firmly supported by the data obtained in our experiments.

**2. Has the statistical analysis been performed appropriately and rigorously?**

**Reviewer #1: No	Reviewer #2: Yes**

In this study, statistical analysis was performed to examine the significant difference of exon combination (Fig 3 in the revised manuscript) and indel frequency (Fig 4 in the revised manuscript) between normal Jk^a^ group and Jk(a+^w^) group. The criteria for subject selection were clearly explained in the manuscript. Difference was deemed significant only when *p*<0.05. Therefore the statistical analysis is reasonable and the results are convincing.

**3. Have the authors made all data underlying the findings in their manuscript fully available?**

**Reviewer #1: Yes	Reviewer #2: Yes**

We thank the reviewers for their agreement.

**4. Is the manuscript presented in an intelligible fashion and written in standard English?**

**Reviewer #1: Yes	Reviewer #2: Yes**

We thank the reviewers for their agreement.

**Response to Reviewer_1’s comments**

**1. In this study, the authors investigated the role of transcription errors and aberrant splicing in the expression of the Kidd (JK) blood group system, with a particular focus on surface expression of the JKa antigen on reticulocytes. Using PacBio long-read sequencing, they sequenced SLC14A1 cDNA from 18 blood donors, of whom 8 were serologically typed as JK(a+) and 10 exhibited weakened JKa expression. By comparing publicly available mRNA sequences with their own data, the authors identified 15 potential exonic regions. For each sample, they quantified the number of reads harboring these regions as evidence for the presence of different transcript isoforms. In addition, they transfected HEK293T cells with plasmids encoding SLC14A1 open reading frames and compared surface JKa antigen availability with total JK protein expression. The aim of this experiment was to determine whether all expressed proteins exhibited the JKa antigen, thereby assessing whether factors other than amino acid sequence —such as spatial conformation—could influence JKa antigenicity.**

We thank the reviewer for his/her time and efforts in reading our manuscript.

**2. Overall, the biological mechanism investigated in this study is interesting and, to my knowledge, has not been previously explored. This work follows an earlier study by the same authors in which they investigated a promoter polymorphism in the JK*01W.06 allele to explain the weak JK(a+w) phenotype. While the overall logic of this follow-up study is sound, the rationale for focusing on transcript-level variation in weak JK(a+w) samples should be better explained, particularly given the broad readership of a non-transfusion journal such as PLOS ONE.**

We thank the reviewer for his/her agreement with the topic of our study and appreciate his/her attention to our previous work (PMID: 40484950).

We agree that a study for publishing in *PLOS ONE* should be made understandable to a broad readership. As we mentioned in the introduction, changes of mRNA structure have been widely investigated not only in antigen presentation, but also in function alterations of various proteins in the studies of disease and development. In this manuscript we have also cited some of our published works on the influences of mRNA structure alteration in the field of not only transfusion (PMID: 35138639), but also cancer research (PMID: 32198183).

Therefore, we believe the methods and the results presented in this manuscript may also help researchers in the fields other than transfusion.

**3. The authors claim that their data provide evidence that transcription errors and aberrant splicing play an important role in the weak JKa phenotype. However, I have strong concerns regarding both the data and the conclusions drawn from them.**

**First, with respect to transcription errors, it is well established that transcription by RNA polymerase II is highly accurate (e.g. Fritsch et al., PNAS, 2020) and, as the authors note themselves, transcription errors are stochastic. Although such errors may exhibit sequence-context biases, they do not generate reproducible or structured transcript variants. Consequently, recurrent SNVs or indels observed at appreciable frequencies are unlikely to originate from transcriptional misincorporation, but rather from reverse transcription, PCR amplification, or sequencing artifacts. As the authors did not employ a molecular strategy that would allow discrimination of true transcription errors from technical artifacts, the analysis of the c.130 variant is, in my view, not valid.**

In the work cited by the reviewer (PMID: 33443141), the researchers focused their study on the transcription errors that were caused by mutagen-induced DNA damage. Fidelity of RNA polymerase II (POL II) was not purposely investigated hence no relevant conclusions were reached. However, one of their results (Fig. 2C in PMID: 33443141) showed that deletion of *RPB9* gene, which encodes one of the POL II subunits, resulted in reduced transcription fidelity.

Maintenance of transcriptional fidelity depends on the stability of POL II complex composed of 12 subunits that may be dysregulated under certain conditions, such as cancers and other diseases (PMID: 37132022). In one of our published works (PMID: 32198183) that is cited in this manuscript, we reported that downregulation of *RPB9*, the gene encoding subunit POLR2I, correlated with higher rate of transcription errors in liver cancer. Because gene expression regulation is dynamic (PMID: 41588170), our data in this manuscript suggest a potential correlation between *SLC14A1* transcription errors and fluctuation of POL II stability, either transient or for long term.

To exclude technical artifact due to PCR amplification and sequencing process, we performed PacBio analysis with SLC14A1 fragments amplified from sequence-confirmed plasmids. Results showed the mismatch rate was lower than 5%. The data have been included in Table S1 in the revised manuscript.

Reverse transcription (RT)-related artifacts, if there are, should be universally and evenly affect all the research subjects. However, when we look through the results in S1 Table and Fig 4 in the revised manuscript, mismatch patterns are different among the research subjects, hence we think that the RT-related artifacts can be excluded.

**3. Second, with respect to aberrant or alternative splicing, it is indeed plausible that splicing variation could contribute to weakening of the JKa antigen. However, the data do not show a clear shift in dominant isoform usage between weak and “normal” JK(a+) samples. Although the authors report significant differences in the presence of PER-6 and PER-7 between JK(a+) and JK(a+w) samples, the observation that transcripts from N5 and N7 samples also harbor these PERs argues against a causal role of these regions in weakening JKa expression. Furthermore, while inter-individual differences in splice isoform abundance can occur, the presence of different dominant isoforms within the same population and tissue, in the absence of genetic variants affecting splicing, is not the expected default. Such patterns require careful validation to exclude technical artifacts, particularly in light of the variable read depth and limited sample size. Given that the authors state that “the integrity of the SLC14A1 gene was confirmed via PacBio long-read sequencing of genomic DNA,” a clearer link between genetic variation and transcript structure should be established.**

As the reviewer mentioned, our statistical analysis shows PER-6 and PER-7 skipping rates are significantly higher in normal JKa group, even though N5 and N7 were included. The results clearly indicate SLC14A1 protein isoform ratios are different between normal and weak JKa groups. Different protein isoforms may present different antigenic features due to changes of amino acid sequence, post-translational modification and protein conformation. In this study, we have also found that inclusion of PER-6 and PER-7 may increase the risk of transcription errors at 130A position that will cause frameshift or early termination in protein translation. Therefore, under the premise of equivalent gene expression, different SLC14A1 isoform ratios may lead to different detection thresholds in blood serological typing when a standard anti-serum is used in clinical practice.

We thank the reviewer for pointing out N5 and N7 subjects for discussion. Actually in N7 subjects, the proportions of the reads containing PER-6 or PER-7 are 55% and 53% respectively, much lower than the average percentage in weak JK^a^ group. Interestingly, “A” insertions at 130A position were observed in almost all the PER-6 containing transcripts (S1 Table in revised manuscript). However, possibly because of the homozygous JK^a^ genotype, the individual N7 still demonstrated normal Jk^a^ phenotype due to “double dose” effect.

In N5 subject, we found about 400 reads containing PER-6 and PER-7 also contained PER-3B and PER-3C that introduced novel ATG start codons (Table 1). Further investigation need to be done to explain the significance hence we did not discuss in this manuscript. If we deduct these 400 reads, the proportion will be about 60%, also much lower than the average percentage in weak JKa group.

Like the maintenance of transcription fidelity of POL II, RNA splicing is highly dynamic depending on the regulations of the spliceosome complex under various conditions (PMID: 38641465). Therefore, ratio of different SLC14A1 isoforms may keep changing within the same population and tissue in the absence of genetic variants.

**4. Third, as presented, the results from the cell transfection, western blot, and immunostaining experiments are not fully convincing, as the authors did not control for the immunoglobulin class of the antibodies used (e.g., IgG versus IgM). Consequently, the observed differences between total SLC14A1 protein detection and JKa antigen detection could reflect differences in antibody avidity rather than differences in protein spatial conformation, as suggested by the authors.**

At the same time when we perform transfection with *SLC14A1* plasmids to overexpress the protein in 293T cells, we also transfected blank plasmids to prepare control samples. Therefore, in western and immunostaining we have appropriate negative control samples to exclude the possibility of non-specific staining.

The Pelikloon anti-Jka IgM (PMID: 9259833) that we used in this study is commonly used in blood typing in clinical practice. Its specificity has been well tested hence differences in antibody avidity should not be a concern.

**5. In addition to these major points, several methodological aspects remain unclear. For example, it is not explained why only forward reads were analyzed. Moreover, given the use of long-read sequencing, it is unclear why the authors focused on exon-level rather than full-length transcript-level analyses. A transcript-level approach would have allowed restriction of the analysis to JK(a) alleles only. As currently presented, Table 1 and Figure S3 appear to include JK(b) transcripts, which may obscure genuine shifts in JK(a+) isoform abundance between weak and “normal” alleles.**

In this study double-strand PCR products were input for PacBio sequencing. Different from Sanger sequencing, in PacBio each PCR fragment will theoretically generate two sequencing data, forward and reverse, both provided the same information. Therefore, we extracted forward reads as valid data for analysis. We have explained the valid reads extraction in detail in the original version of the manuscript.

Using the sequential filtering methods that we carefully described in this manuscript, we could also extract *JK^a^* allele-encoded transcripts for analysis. For example, in this work we have evaluated exon combination patterns not only in total *JK* transcripts (Fig 3A in the revised manuscript), but also in *JK^a^* transcripts alone (Fig 3B in the revised manuscript). Hence, we don't think in our analysis *JK^b^* transcripts may obscure genuine shifts in JK(a+) isoform abundance between weak and “normal” alleles.

In addition, sequential filtering with fragmented sequences helped us to improve the resolution and accuracy of the detailed changes of target sequences that might be overlooked in full-length transcript-level high-throughput analysis.

**6. Furthermore, based on the data presented, all phenotypes described as “weak” should in fact be referred to as “null” phenotypes (JK(a−)). The authors should clearly state that most of these weak phenotypes appear as null by routine serological testing, mainly/especially in han population, but show agglutination only after protease treatment. It would also be important to distinguish true null phenotypes from weak phenotypes, as sample W3 would be expected to represent a true null, given the absence of PER-11 from its transcripts.**

The blood samples involved in this study were determined as "weak" or "normal" JKa according to the clinical standard of serological testing in Shenzhen Blood Center. In most subjects *JK^a^* PER-11 containing transcripts have been detected, indicating Jk^a^ antigens, no matter whether the levels were low or high, were expressed. We agree with the reviewer that W3 seemed to present a real null phenotype, however, due to the confirmed integrity of *SLC14A1* gene (S1 Fig in the revised manuscript), stringent standards for JK^null^ diagnosis (PMID: 30820956), lack of gene expression level quantification as well as the dynamic features of RNA splicing process, we think it not time to name it as "null" phenotype.

**Response to Reviewer_2’s comments**

**1. The manuscript “Alterations of SLC14A1 mRNA structure are frequently detected in weak Jka presenting donors” from Liang et al. is an interesting examination of alternate splicing in SLC14A1 as it pertains to antigen presentation in the Kidd blood group.**

**Whilst the manuscript is reasonably well written, it was quite difficult to follow at times.**

We thank the reviewer for his/her time and efforts in reading our manuscript.

**2. Figure S3 – this ‘table’ is far too big for a figure, and when zoomed in enough to be readable, is impossible to follow.**

We have changed this supplementary information from a figure (S3 Fig in the original version) to an Excel file (S1 Table in the revised version) to make the data easier to follow.

**3. Table 1 - I am unclear on exactly how the number of valid reads was determined. In the body of the manuscript it states: “As expected about half of the total reads were extracted as valid reads because double strand PCR products were input for PacBio sequencing” however, when adding up the number of reads across each PER for the samples, the number of valid reads appears to be ¼ of the reads listed (and total reads are about ½ the reads listed)**

In this study double-strand PCR products were input for PacBio sequencing. Different from Sanger sequencing, in PacBio each PCR fragment will theoretically generate two sequencing data, forward and reverse, both provided the same information. Therefore, we used verification sequences of PER-2 and PER-12 (Fig 2B in the revised manuscript) to extract forward reads as valid data for analysis. Theoretically the valid reads should be half of the total reads. However, due to artifact of PCR amplification (please refer to plasmid-based sequencing data in S1 Table in the revised manuscript) and error rate of PacBio sequencing (~0.1%), the extracted valid reads were slightly fewer than half of the total reads.

In this manuscript we claimed that not all exons evenly included in all transcripts, leading to translation of various protein isoforms. Particularly, PER-6 and PER-7 inclusion patterns showed a big difference between "normal" and "weak" groups. For example, in N1 subject, only 608 transcripts contain PER-6, fewer than 1/2 of 1476 valid reads, about 1/5 of 3005 total reads (Table 1 in the revised manuscript).

**4. Figure 1B – the pacbio sequencing results are already included in table 1 – is there an error in the total reads for W1 in table 1 or figure 1B? (figure 1B says 6713, table 1 says 6613). Furthermore – DNA ?genotyping/haplotypes were reported using only four nucleotide positions – a full genotype (based on alleles reported in the ISBT blood group database) would have been good to include, given that some of the nucleotide positions are associated with normal, weak or null alleles. This would have also been interesting in the context of indels at 130A regions.**

We thank the reviewer for pointing out the mistake in original manuscript. Total reads of subject W1 should be 6713. We have corrected the error in Table 1 and S1 Table of the revised manuscript.

In this study, we selected 4 well known SNP sites in *SLC14A1* gene to form a haplotype and used it to differentiate SLC14A1 transcripts derived from different alleles in the sequential filtering analysis. By doing this we may analyse the detailed changes of transcripts encoded by different alleles of each research subject.

**5. Figure 3A – nucleotides are cut off at the top of the chromatogram – it is unclear where the “C” deletion in W2 mRNA is depicted in the chromatogram.**

In Fig 3A of the original version (Fig 4A in the revised manuscript), the minor peaks in the Sanger chromatogram of W3 mRNA represent sequences of transcripts with “C” deletion. We have made further explanation in the figure legend in the revised manuscript.

**6. Minor errors representing antigen nomenclature throughout – eg. Jk(a) antigen and Jk(b) antigen, or with the antigen designation as superscript, whereas some parts of text have Jka Jkb antigen, some sections where the +w is not superscript, and gene names not italicised in various parts of the text.**

We thank the reviewer for pointing out the careless mistakes. We have checked and corrected the errors in the revised manuscript

**7. Figure 1 a/b should really be their own figure as they form part of the materials and methods rather than results.**

We thank the reviewer for the constructive advice and have split Fig 1 in original version into two figures (Figs 1 and Fig 2 in the revised manuscript).

**8. Figure 1c is not particularly informative as it does not graphically represent the exonic/transcript makeup of the SLC14A1 gene, nor the read direction.**

In this study, we extracted sequence of SLC14A1 gene (~40kb) from Chr 18 (NCBI #NC_000018.10) to generate a reference sequence to facilitate the alignment analysis and result description/explanation in the manuscript. We add this schematic diagram (Fig 2A in the revised manuscript) to depict the location of this region in Chr 18 so that if any researchers feel interested in our results, they can easily verify/repeat the analysis in our manuscript. Therefore, we think this figure is necessary in the manuscript.
