# Supplementary material for: Alterations of SLC14A1 mRNA structure are frequently detected in weak Jka presenting donors
Source: PLoS One. 2026 Oct 14;21(10):e0360437. doi: 10.1371/journal.pone.0360437 (PMC13667791; doi:10.1371/journal.pone.0360437)
Supplement: Supplementary file 3 [file pone.0360437.s012.docx]

**Response to Editor’s comments**

**1. It was examined by the same referees who had review the original submission. As you can see from their comments, one referee continues to have a serious concern; namely, about the possibility of reverse transcription artifacts. Considering the somewhat extra-ordinariness of the RNA polymerase-related mechanism that the manuscript has proposed to explain some of its findings, it is important to reduce the likelihood that the findings are reverse transcription (RT) artifacts. As you know, multiple studies have noted such artifacts (e.g., PMID 34183059). I note your rebuttal that RT artifacts, if true, should have occurred for all subjects of the study. But, inter-sample difference for RT artifacts is theoretically possible (PMID 36990512), and one can raise a similar concern for a polymerase error-based mechanism. Additional data to rule out RT artifacts is therefore important to support the manuscript's findings.**

We greatly appreciate Dr. Patnaik's constructive comments and agree that additional data to rule out RT artifacts is important to support the manuscript's findings. Following the suggestions, we designed and *in vitro* synthesized a RNA template and subjected it to RT-PCR to amplify *SLC4A1* fragments for PacBio sequencing analysis. The results demonstrated that RT-PCR processes could really generated artifacts whereas the mismatching rates and indel rates were much lower than that we observed in the research subjects. Hence the RT-PCR artifacts do not significantly affect the conclusions in this study. We have added the data (S1 Table and S3 Fig) and the suggested references in the revised manuscript.

**2. Regarding the data in figure 5, just one type of assay with one cell-line and a couple of antibodies is not sufficient to come to a conclusion. I hope you can provide additional data to support it, or you can choose to remove this aspect of the study from the manuscript as it is currently not central to the manuscript.**

We agree that the results obtained with one cell-line and a couple of antibodies are not sufficient to support the conclusions in this study. Therefore, in the revised manuscript we have removed this part of the work (Fig 5 in the previous manuscript) according to Dr. Patnaik's kind advices.

**2. Finally, in any revision, please ensure that you incorporate in the manuscript the various rebuttals that you wrote in response to the the first review.**

We sincerely apologize for not incorporating our replies to the reviewers's comments in the previously revised manuscript. In the newly revised manuscript, we have added the discussions upon the reviewers' concerns, such as the novelty of the methods in this study and the dynamic regulations of transcription fidelity and spliceosome activity. We have also cited relevant publications in the revised manuscript.

**Response to Reviewer_1’s comments**

**1. The authors have responded to all reviewer comments; however, only minimal changes were made to the manuscript itself. Many of the questions raised during the first review round are likely the same questions that readers will ask when evaluating the study. Therefore, several of the explanations provided in the rebuttal should, in my opinion, be incorporated directly into the manuscript rather than remaining only in the response letter.**

We sincerely apologize for not incorporating our replies to the reviewers's comments in the previously revised manuscript. In the newly revised manuscript, we have added the discussions upon the reviewers' concerns, such as the novelty of the methods in this study and the dynamic regulations of transcription fidelity and spliceosome activity. We have also cited relevant publications in the revised manuscript.

**2. In the first round, I raised a major concern regarding one of the central conclusions of this work, namely the proposed role of RNA polymerase II transcription errors in weakening JK(a) expression. I agree that sequencing sequence-confirmed plasmids using PacBio can help assess potential PCR amplification and sequencing errors, although a reported mismatch rate of 5% appears unexpectedly high for PacBio sequencing and itself raises concerns. Nevertheless, this control does not address the major confounding factor in cDNA-based studies, namely reverse transcription–related errors.**

**Importantly, RNA polymerase II has an estimated error rate of approximately 3.9 × 10⁻⁶ per base, with insertion error rates around 7.4 × 10⁻⁷ per base (Gout et al., 2017; PMID: 29062891). Although the product documentation for SuperScript III reverse transcriptase does not provide a precise numerical error rate, Thermo Fisher reports that newer systems such as the SuperScript IV One-Step RT-PCR System achieve lower overall error rates due to the use of a high-fidelity DNA polymerase during the PCR step, with reported rates in the range of 0.5–1.5 × 10⁻³ per base (as reported by the manufacturer (https://documents.thermofisher.com/TFS-Assets/BID/Reference-Materials/advanced-analysis-superscript iv-one-step-rt-pcr-system-white-paper.pdf). Thus, the probability that the observed indels were introduced during reverse transcription is several orders of magnitude higher than the probability that they originated from true transcriptional errors. As noted by Gout et al., standard cDNA libraries are therefore expected to be dominated by reverse transcription errors rather than genuine RNA polymerase II transcription errors.**

**Because the authors did not include a molecular strategy to distinguish true transcriptional errors from reverse transcription artifacts (such as UMIs, independent RT replicates, or direct RNA sequencing), the conclusion that “our results have demonstrated that alternative splicing and transcription error are important risk factors for weak JKa antigen presentation” is not sufficiently supported by the data and, in my view, remains invalid.**

We sincerely appreciate the reviewer's efforts in explaining the big concern about the reliability of the transcription error analysis in this study. We agree that additional experiments should be conducted to exclude the RT-PCR artifacts. Therefore, we designed and synthesized a RNA template using T7 *in vitro* transcription system. Then we performed RT-PCR to amplify *SLC14A1* fragments from the RNA and subjected the PCR products to PacBio sequencing analysis. In the RNA template there were two gene cassettes containing tandem connected exons, PER-6 and PER-7. One cassette carried 130A-222A-326C haplotype of SLC14A1 ORF and the other cassette carried 130G-222C-326T haplotype so that we could distinguish the two regions in the PacBio sequencing reads. By doing this we could also compare the artifact rates occurring at the two positions that were intensively investigated in this study.

As shown in S3 Fig in the revised manuscript, the indel rates in the 6-nucleotide region were much lower than that observed in the research subjects (Fig 4 in the revised manuscript), indicating RT-PCR artifacts can be ignored when analysing the transcription errors in this study.

Detailed information of the experiments, including the design, the results and summary as well as the discussion has been included in the revised manuscript.
