# Supplementary material for: Alterations of SLC14A1 mRNA structure are frequently detected in weak Jka presenting donors
Source: PLoS One. 2026 Oct 14;21(10):e0360437. doi: 10.1371/journal.pone.0360437 (PMC13667791; doi:10.1371/journal.pone.0360437)
Supplement: Supplementary file 4 [file pone.0360437.s013.docx]

**Response to Editor’s comments**

**1. Thank you for submitting the revised manuscript with additional data and text to address the concerns raised in the previous review. I was unable to get any of the two referees who had examined the previous version of the manuscript to re-examine the revised manuscript. But I have gone through the manuscript changes and feel that they reasonably address the major concerns that were raised in the last review. However, I request you to edit the manuscript's abstract to add a statement effectively indicating that "additional experiments suggest that RT artifacts were unlikely the cause of these observations.**

We thank Dr. Patnaik for taking time to examine our manuscript and greatly appreciate his approval of the revisions. According to his kind instruction, we have edited the abstract by adding "Technical artifacts were examined using synthesized RNA templates and the results suggested that artifacts generated in the reverse transcription and PCR amplification (RT-PCR) processes were unlikely the causes of these observations".
