# Supplementary material for: Alterations of SLC14A1 mRNA structure are frequently detected in weak Jka presenting donors
Source: PLoS One. 2026 Oct 14;21(10):e0360437. doi: 10.1371/journal.pone.0360437 (PMC13667791; doi:10.1371/journal.pone.0360437)
Supplement: Peer review history

# Peer review: Decision Letter 0

**DOI:** 10.1371/journal.pone.0360437.r001

**Authors:** Santosh Patnaik

20 Feb 2026

Dear Dr. REN,

Thank you for submitting your manuscript to PLOS ONE. After careful consideration, we feel that it has merit but does not fully meet PLOS ONE’s publication criteria as it currently stands. Therefore, we invite you to submit a revised version of the manuscript that addresses the points raised during the review process.

Please submit your revised manuscript by Apr 05 2026 11:59PM. If you will need more time than this to complete your revisions, please reply to this message or contact the journal office at plosone@plos.org. When you're ready to submit your revision, log on to https://www.editorialmanager.com/pone/ and select the 'Submissions Needing Revision' folder to locate your manuscript file.

- A letter that responds to each point raised by the academic editor and reviewer(s). You should upload this letter as a separate file labeled 'Response to Reviewers'.
- A marked-up copy of your manuscript that highlights changes made to the original version. You should upload this as a separate file labeled 'Revised Manuscript with Track Changes'.
- An unmarked version of your revised paper without tracked changes. You should upload this as a separate file labeled 'Manuscript'.

If you would like to make changes to your financial disclosure, please include your updated statement in your cover letter. Guidelines for resubmitting your figure files are available below the reviewer comments at the end of this letter.

If applicable, we recommend that you deposit your laboratory protocols in protocols.io to enhance the reproducibility of your results. Protocols.io assigns your protocol its own identifier (DOI) so that it can be cited independently in the future. For instructions see: https://journals.plos.org/plosone/s/submission-guidelines#loc-laboratory-protocols. Additionally, PLOS ONE offers an option for publishing peer-reviewed Lab Protocol articles, which describe protocols hosted on protocols.io. Read more information on sharing protocols at https://plos.org/protocols?utm_medium=editorial-email&utm_source=authorletters&utm_campaign=protocols.

We look forward to receiving your revised manuscript.

Kind regards,

Santosh K. Patnaik, MD, PhD

Academic Editor

PLOS One

Journal Requirements:

When submitting your revision, we need you to address these additional requirements.

1. Please ensure that your manuscript meets PLOS ONE's style requirements, including those for file naming. The PLOS ONE style templates can be found at

https://journals.plos.org/plosone/s/file?id=wjVg/PLOSOne_formatting_sample_main_body.pdf and

https://journals.plos.org/plosone/s/file?id=ba62/PLOSOne_formatting_sample_title_authors_affiliations.pdf

2. Thank you for stating the following financial disclosure:

“SL received following grants:

1) The Natural Science Foundation of Shenzhen Municipality (No. JCYJ20230807154000002)

2) Guangdong Medical Foundation (No. B2025582)

3) Shenzhen Key Medical Discipline Construction Fund (No. SZXK070)

4) Sanming Project of Medicine in Shenzen Municipality (No.SZSM202311032)”

Please state what role the funders took in the study.  If the funders had no role, please state: "The funders had no role in study design, data collection and analysis, decision to publish, or preparation of the manuscript." If this statement is not correct you must amend it as needed.

Please include this amended Role of Funder statement in your cover letter; we will change the online submission form on your behalf.

3. Please note that funding information should not appear in the Acknowledgments section or other areas of your manuscript. We will only publish funding information present in the Funding Statement section of the online submission form. Please remove any funding-related text from the manuscript.

4. Please include captions for your Supporting Information files at the end of your manuscript, and update any in-text citations to match accordingly. Please see our Supporting Information guidelines for more information: http://journals.plos.org/plosone/s/supporting-information.

5. If the reviewer comments include a recommendation to cite specific previously published works, please review and evaluate these publications to determine whether they are relevant and should be cited. There is no requirement to cite these works unless the editor has indicated otherwise.

**Additional Editor Comments:**

The manuscript was examined by two referees, one of whom has suggested rejection because of a number of serious concerns. I agree with most of the concerns, but I would like to give you the opportunity to address them by providing additional data, revisions to how interpretations/conclusions, and/or rebuttals.

[Note: HTML markup is below. Please do not edit.]

Reviewers' comments:

Reviewer's Responses to Questions

**Comments to the Author**

1. Is the manuscript technically sound, and do the data support the conclusions?

Reviewer #1: No

Reviewer #2: Partly

**********

2. Has the statistical analysis been performed appropriately and rigorously?

Reviewer #1: No

Reviewer #2: Yes

**********

3. Have the authors made all data underlying the findings in their manuscript fully available?

The PLOS Data policy

Reviewer #1: Yes

Reviewer #2: Yes

**********

4. Is the manuscript presented in an intelligible fashion and written in standard English?

Reviewer #1: Yes

Reviewer #2: Yes

**********

Reviewer #1: In this study, the authors investigated the role of transcription errors and aberrant splicing in the expression of the Kidd (JK) blood group system, with a particular focus on surface expression of the JKa antigen on reticulocytes. Using PacBio long-read sequencing, they sequenced SLC14A1 cDNA from 18 blood donors, of whom 8 were serologically typed as JK(a+) and 10 exhibited weakened JKa expression. By comparing publicly available mRNA sequences with their own data, the authors identified 15 potential exonic regions. For each sample, they quantified the number of reads harboring these regions as evidence for the presence of different transcript isoforms. In addition, they transfected HEK293T cells with plasmids encoding SLC14A1 open reading frames and compared surface JKa antigen availability with total JK protein expression. The aim of this experiment was to determine whether all expressed proteins exhibited the JKa antigen, thereby assessing whether factors other than amino acid sequence—such as spatial conformation—could influence JKa antigenicity.

Overall, the biological mechanism investigated in this study is interesting and, to my knowledge, has not been previously explored. This work follows an earlier study by the same authors in which they investigated a promoter polymorphism in the JK*01W.06 allele to explain the weak JK(a+w) phenotype. While the overall logic of this follow-up study is sound, the rationale for focusing on transcript-level variation in weak JK(a+w) samples should be better explained, particularly given the broad readership of a non-transfusion journal such as PLOS ONE.

The authors claim that their data provide evidence that transcription errors and aberrant splicing play an important role in the weak JKa phenotype. However, I have strong concerns regarding both the data and the conclusions drawn from them.

First, with respect to transcription errors, it is well established that transcription by RNA polymerase II is highly accurate (e.g. Fritsch et al., PNAS, 2020) and, as the authors note themselves, transcription errors are stochastic. Although such errors may exhibit sequence-context biases, they do not generate reproducible or structured transcript variants. Consequently, recurrent SNVs or indels observed at appreciable frequencies are unlikely to originate from transcriptional misincorporation, but rather from reverse transcription, PCR amplification, or sequencing artifacts. As the authors did not employ a molecular strategy that would allow discrimination of true transcription errors from technical artifacts, the analysis of the c.130 variant is, in my view, not valid.

Second, with respect to aberrant or alternative splicing, it is indeed plausible that splicing variation could contribute to weakening of the JKa antigen. However, the data do not show a clear shift in dominant isoform usage between weak and “normal” JK(a+) samples. Although the authors report significant differences in the presence of PER-6 and PER-7 between JK(a+) and JK(a+w) samples, the observation that transcripts from N5 and N7 samples also harbor these PERs argues against a causal role of these regions in weakening JKa expression. Furthermore, while inter-individual differences in splice isoform abundance can occur, the presence of different dominant isoforms within the same population and tissue, in the absence of genetic variants affecting splicing, is not the expected default. Such patterns require careful validation to exclude technical artifacts, particularly in light of the variable read depth and limited sample size. Given that the authors state that “the integrity of the SLC14A1 gene was confirmed via PacBio long-read sequencing of genomic DNA,” a clearer link between genetic variation and transcript structure should be established.

Third, as presented, the results from the cell transfection, western blot, and immunostaining experiments are not fully convincing, as the authors did not control for the immunoglobulin class of the antibodies used (e.g., IgG versus IgM). Consequently, the observed differences between total SLC14A1 protein detection and JKa antigen detection could reflect differences in antibody avidity rather than differences in protein spatial conformation, as suggested by the authors.

In addition to these major points, several methodological aspects remain unclear. For example, it is not explained why only forward reads were analyzed. Moreover, given the use of long-read sequencing, it is unclear why the authors focused on exon-level rather than full-length transcript-level analyses. A transcript-level approach would have allowed restriction of the analysis to JK(a) alleles only. As currently presented, Table 1 and Figure S3 appear to include JK(b) transcripts, which may obscure genuine shifts in JK(a+) isoform abundance between weak and “normal” alleles.

Furthermore, based on the data presented, all phenotypes described as “weak” should in fact be referred to as “null” phenotypes (JK(a−)). The authors should clearly state that most of these weak phenotypes appear as null by routine serological testing, mainly/especially in han population, but show agglutination only after protease treatment. It would also be important to distinguish true null phenotypes from weak phenotypes, as sample W3 would be expected to represent a true null, given the absence of PER-11 from its transcripts.

Reviewer #2: The manuscript “Alterations of SLC14A1 mRNA structure are frequently detected in weak Jka presenting donors” from Liang et al. is an interesting examination of alternate splicing in SLC14A1 as it pertains to antigen presentation in the Kidd blood group.

Whilst the manuscript is reasonably well written, it was quite difficult to follow at times.

Major concerns:

Figure S3 – this ‘table’ is far too big for a figure, and when zoomed in enough to be readable, is impossible to follow.

Table 1 - I am unclear on exactly how the number of valid reads was determined. In the body of the manuscript it states: “As expected about half of the total reads were extracted as valid reads because double strand PCR products were input for PacBio sequencing” however, when adding up the number of reads across each PER for the samples, the number of valid reads appears to be ¼ of the reads listed (and total reads are about ½ the reads listed)

Figure 1B – the pacbio sequencing results are already included in table 1 – is there an error in the total reads for W1 in table 1 or figure 1B? (figure 1B says 6713, table 1 says 6613). Furthermore – DNA ?genotyping/haplotypes were reported using only four nucleotide positions – a full genotype (based on alleles reported in the ISBT blood group database) would have been good to include, given that some of the nucleotide positions are associated with normal, weak or null alleles. This would have also been interesting in the context of indels at 130A regions.

Figure 3A – nucleotides are cut off at the top of the chromatogram – it is unclear where the “C” deletion in W2 mRNA is depicted in the chromatogram.

General concerns:

Minor errors representing antigen nomenclature throughout – eg. Jk(a) antigen and Jk(b) antigen, or with the antigen designation as superscript, whereas some parts of text have Jka Jkb antigen, some sections where the +w is not superscript, and gene names not italicised in various parts of the text.

Figure 1 a/b should really be their own figure as they form part of the materials and methods rather than results.

Figure 1c is not particularly informative as it does not graphically represent the exonic/transcript makeup of the SLC14A1 gene, nor the read direction.

**********

what does this mean?). If published, this will include your full peer review and any attached files.

If you choose “no”, your identity will remain anonymous but your review may still be made public.

**Do you want your identity to be public for this peer review?**  For information about this choice, including consent withdrawal, please see our Privacy Policy

Reviewer #1: No

Reviewer #2: No

**********

[NOTE: If reviewer comments were submitted as an attachment file, they will be attached to this email and accessible via the submission site. Please log into your account, locate the manuscript record, and check for the action link "View Attachments". If this link does not appear, there are no attachment files.]

To ensure your figures meet our technical requirements, please review our figure guidelines: https://journals.plos.org/plosone/s/figures

You may also use PLOS’s free figure tool, NAAS, to help you prepare publication quality figures: https://journals.plos.org/plosone/s/figures#loc-tools-for-figure-preparation.

NAAS will assess whether your figures meet our technical requirements by comparing each figure against our figure specifications.

# Author comment: Author response to Decision Letter 1

**DOI:** 10.1371/journal.pone.0360437.r002

5 Apr 2026

We have carefully read the reviewers' comments and addressed their concerns point by point in "Response to Reviewers" that has been uploaded for further examination with the revised version of the manuscript. Thank you.

Attachment Submitted filename: Response to Reviewers_PLOS ONE 20260324.docx

# Peer review: Decision Letter 1

**DOI:** 10.1371/journal.pone.0360437.r003

**Authors:** Santosh Patnaik

28 Apr 2026

Dear Dr. REN,

Thank you for submitting your manuscript to PLOS ONE. After careful consideration, we feel that it has merit but does not fully meet PLOS ONE’s publication criteria as it currently stands. Therefore, we invite you to submit a revised version of the manuscript that addresses the points raised during the review process.

It was examined by the same referees who had review the original submission. As you can see from their comments, one referee continues to have a serious concern; namely, about the possibility of reverse transcription artifacts. Considering the somewhat extra-ordinariness of the RNA polymerase-related mechanism that the manuscript has proposed to explain some of its findings, it is important to reduce the likelihood that the findings are reverse transcription (RT) artifacts. As you know, multiple studies have noted such artifacts (e.g., PMID 34183059). I note your rebuttal that RT artifacts, if true, should have occurred for all subjects of the study. But, inter-sample difference for RT artifacts is theoretically possible (PMID 36990512), and one can raise a similar concern for a polymerase error-based mechanism. Additional data to rule out RT artifacts is therefore important to support the manuscript's findings.

Regarding the data in figure 5, just one type of assay with one cell-line and a couple of antibodies is not sufficient to come to a conclusion. I hope you can provide additional data to support it, or you can choose to remove this aspect of the study from the manuscript as it is currently not central to the manuscript.

Finally, in any revision, please ensure that you incorporate in the manuscript the various rebuttals that you wrote in response to the the first review.

Please submit your revised manuscript by Jun 12 2026 11:59PM. If you will need more time than this to complete your revisions, please reply to this message or contact the journal office at plosone@plos.org. When you're ready to submit your revision, log on to https://www.editorialmanager.com/pone/ and select the 'Submissions Needing Revision' folder to locate your manuscript file.

- A letter that responds to each point raised by the academic editor and reviewer(s). You should upload this letter as a separate file labeled 'Response to Reviewers'.
- A marked-up copy of your manuscript that highlights changes made to the original version. You should upload this as a separate file labeled 'Revised Manuscript with Track Changes'.
- An unmarked version of your revised paper without tracked changes. You should upload this as a separate file labeled 'Manuscript'.

If you would like to make changes to your financial disclosure, please include your updated statement in your cover letter. Guidelines for resubmitting your figure files are available below the reviewer comments at the end of this letter.

If applicable, we recommend that you deposit your laboratory protocols in protocols.io to enhance the reproducibility of your results. Protocols.io assigns your protocol its own identifier (DOI) so that it can be cited independently in the future. For instructions see: https://journals.plos.org/plosone/s/submission-guidelines#loc-laboratory-protocols. Additionally, PLOS ONE offers an option for publishing peer-reviewed Lab Protocol articles, which describe protocols hosted on protocols.io. Read more information on sharing protocols at https://plos.org/protocols?utm_medium=editorial-email&utm_source=authorletters&utm_campaign=protocols.

As the corresponding author, your ORCID iD is verified in the submission system and will appear in the published article. PLOS supports the use of ORCID, and we encourage all coauthors to register for an ORCID iD and use it as well. Please encourage your coauthors to verify their ORCID iD within the submission system before final acceptance, as unverified ORCID iDs will not appear in the published article. *Only*  the individual author can complete the verification step; PLOS staff *cannot*  verify ORCID iDs on behalf of authors.

We look forward to receiving your revised manuscript.

Kind regards,

Santosh K. Patnaik, MD, PhD

Academic Editor

PLOS One

Journal Requirements:

If the reviewer comments include a recommendation to cite specific previously published works, please review and evaluate these publications to determine whether they are relevant and should be cited. There is no requirement to cite these works unless the editor has indicated otherwise.

[Note: HTML markup is below. Please do not edit.]

Reviewers' comments:

Reviewer's Responses to Questions

**Comments to the Author**

Reviewer #1: (No Response)

Reviewer #2: All comments have been addressed

**********

2. Is the manuscript technically sound, and do the data support the conclusions?

Reviewer #1: No

Reviewer #2: Yes

**********

3. Has the statistical analysis been performed appropriately and rigorously?

Reviewer #1: No

Reviewer #2: Yes

**********

4. Have the authors made all data underlying the findings in their manuscript fully available?

The PLOS Data policy

Reviewer #1: Yes

Reviewer #2: Yes

**********

5. Is the manuscript presented in an intelligible fashion and written in standard English?

Reviewer #1: Yes

Reviewer #2: Yes

**********

Reviewer #1: The authors have responded to all reviewer comments; however, only minimal changes were made to the manuscript itself. Many of the questions raised during the first review round are likely the same questions that readers will ask when evaluating the study. Therefore, several of the explanations provided in the rebuttal should, in my opinion, be incorporated directly into the manuscript rather than remaining only in the response letter.

In the first round, I raised a major concern regarding one of the central conclusions of this work, namely the proposed role of RNA polymerase II transcription errors in weakening JK(a) expression. I agree that sequencing sequence-confirmed plasmids using PacBio can help assess potential PCR amplification and sequencing errors, although a reported mismatch rate of 5% appears unexpectedly high for PacBio sequencing and itself raises concerns. Nevertheless, this control does not address the major confounding factor in cDNA-based studies, namely reverse transcription–related errors.

Importantly, RNA polymerase II has an estimated error rate of approximately 3.9 × 10⁻⁶ per base, with insertion error rates around 7.4 × 10⁻⁷ per base (Gout et al., 2017; PMID: 29062891). Although the product documentation for SuperScript III reverse transcriptase does not provide a precise numerical error rate, Thermo Fisher reports that newer systems such as the SuperScript IV One-Step RT-PCR System achieve lower overall error rates due to the use of a high-fidelity DNA polymerase during the PCR step, with reported rates in the range of 0.5–1.5 × 10⁻³ per base (as reported by the manufacturer (https://documents.thermofisher.com/TFS-Assets/BID/Reference-Materials/advanced-analysis-superscript-iv-one-step-rt-pcr-system-white-paper.pdf). Thus, the probability that the observed indels were introduced during reverse transcription is several orders of magnitude higher than the probability that they originated from true transcriptional errors. As noted by Gout et al., standard cDNA libraries are therefore expected to be dominated by reverse transcription errors rather than genuine RNA polymerase II transcription errors.

Because the authors did not include a molecular strategy to distinguish true transcriptional errors from reverse transcription artifacts (such as UMIs, independent RT replicates, or direct RNA sequencing), the conclusion that “our results have demonstrated that alternative splicing and transcription error are important risk factors for weak JKa antigen presentation” is not sufficiently supported by the data and, in my view, remains invalid.

Reviewer #2: (No Response)

**********

what does this mean?). If published, this will include your full peer review and any attached files.

If you choose “no”, your identity will remain anonymous but your review may still be made public.

**Do you want your identity to be public for this peer review?**  For information about this choice, including consent withdrawal, please see our Privacy Policy

Reviewer #1: No

Reviewer #2: No

**********

[NOTE: If reviewer comments were submitted as an attachment file, they will be attached to this email and accessible via the submission site. Please log into your account, locate the manuscript record, and check for the action link "View Attachments". If this link does not appear, there are no attachment files.]

To ensure your figures meet our technical requirements, please review our figure guidelines: https://journals.plos.org/plosone/s/figures

You may also use PLOS’s free figure tool, NAAS, to help you prepare publication quality figures: https://journals.plos.org/plosone/s/figures#loc-tools-for-figure-preparation.

NAAS will assess whether your figures meet our technical requirements by comparing each figure against our figure specifications.

# Author comment: Author response to Decision Letter 2

**DOI:** 10.1371/journal.pone.0360437.r004

29 Jul 2026

Response to Editor’s comments

1. It was examined by the same referees who had review the original submission. As you can see from their comments, one referee continues to have a serious concern; namely, about the possibility of reverse transcription artifacts. Considering the somewhat extra-ordinariness of the RNA polymerase-related mechanism that the manuscript has proposed to explain some of its findings, it is important to reduce the likelihood that the findings are reverse transcription (RT) artifacts. As you know, multiple studies have noted such artifacts (e.g., PMID 34183059). I note your rebuttal that RT artifacts, if true, should have occurred for all subjects of the study. But, inter-sample difference for RT artifacts is theoretically possible (PMID 36990512), and one can raise a similar concern for a polymerase error-based mechanism. Additional data to rule out RT artifacts is therefore important to support the manuscript's findings.

We greatly appreciate Dr. Patnaik's constructive comments and agree that additional data to rule out RT artifacts is important to support the manuscript's findings. Following the suggestions, we designed and in vitro synthesized a RNA template and subjected it to RT-PCR to amplify SLC4A1 fragments for PacBio sequencing analysis. The results demonstrated that RT-PCR processes could really generated artifacts whereas the mismatching rates and indel rates were much lower than that we observed in the research subjects. Hence the RT-PCR artifacts do not significantly affect the conclusions in this study. We have added the data (S1 Table and S3 Fig) and the suggested references in the revised manuscript.

2. Regarding the data in figure 5, just one type of assay with one cell-line and a couple of antibodies is not sufficient to come to a conclusion. I hope you can provide additional data to support it, or you can choose to remove this aspect of the study from the manuscript as it is currently not central to the manuscript.

We agree that the results obtained with one cell-line and a couple of antibodies are not sufficient to support the conclusions in this study. Therefore, in the revised manuscript we have removed this part of the work (Fig 5 in the previous manuscript) according to Dr. Patnaik's kind advices.

2. Finally, in any revision, please ensure that you incorporate in the manuscript the various rebuttals that you wrote in response to the the first review.

We sincerely apologize for not incorporating our replies to the reviewers's comments in the previously revised manuscript. In the newly revised manuscript, we have added the discussions upon the reviewers' concerns, such as the novelty of the methods in this study and the dynamic regulations of transcription fidelity and spliceosome activity. We have also cited relevant publications in the revised manuscript.

Response to Reviewer_1’s comments

1. The authors have responded to all reviewer comments; however, only minimal changes were made to the manuscript itself. Many of the questions raised during the first review round are likely the same questions that readers will ask when evaluating the study. Therefore, several of the explanations provided in the rebuttal should, in my opinion, be incorporated directly into the manuscript rather than remaining only in the response letter.

We sincerely apologize for not incorporating our replies to the reviewers's comments in the previously revised manuscript. In the newly revised manuscript, we have added the discussions upon the reviewers' concerns, such as the novelty of the methods in this study and the dynamic regulations of transcription fidelity and spliceosome activity. We have also cited relevant publications in the revised manuscript.

2. In the first round, I raised a major concern regarding one of the central conclusions of this work, namely the proposed role of RNA polymerase II transcription errors in weakening JK(a) expression. I agree that sequencing sequence-confirmed plasmids using PacBio can help assess potential PCR amplification and sequencing errors, although a reported mismatch rate of 5% appears unexpectedly high for PacBio sequencing and itself raises concerns. Nevertheless, this control does not address the major confounding factor in cDNA-based studies, namely reverse transcription–related errors.

Importantly, RNA polymerase II has an estimated error rate of approximately 3.9 × 10⁻⁶ per base, with insertion error rates around 7.4 × 10⁻⁷ per base (Gout et al., 2017; PMID: 29062891). Although the product documentation for SuperScript III reverse transcriptase does not provide a precise numerical error rate, Thermo Fisher reports that newer systems such as the SuperScript IV One-Step RT-PCR System achieve lower overall error rates due to the use of a high-fidelity DNA polymerase during the PCR step, with reported rates in the range of 0.5–1.5 × 10⁻³ per base (as reported by the manufacturer (https://documents.thermofisher.com/TFS-Assets/BID/Reference-Materials/advanced-analysis-superscript iv-one-step-rt-pcr-system-white-paper.pdf). Thus, the probability that the observed indels were introduced during reverse transcription is several orders of magnitude higher than the probability that they originated from true transcriptional errors. As noted by Gout et al., standard cDNA libraries are therefore expected to be dominated by reverse transcription errors rather than genuine RNA polymerase II transcription errors.

Because the authors did not include a molecular strategy to distinguish true transcriptional errors from reverse transcription artifacts (such as UMIs, independent RT replicates, or direct RNA sequencing), the conclusion that “our results have demonstrated that alternative splicing and transcription error are important risk factors for weak JKa antigen presentation” is not sufficiently supported by the data and, in my view, remains invalid.

We sincerely appreciate the reviewer's efforts in explaining the big concern about the reliability of the transcription error analysis in this study. We agree that additional experiments should be conducted to exclude the RT-PCR artifacts. Therefore, we designed and synthesized a RNA template using T7 in vitro transcription system. Then we performed RT-PCR to amplify SLC14A1 fragments from the RNA and subjected the PCR products to PacBio sequencing analysis. In the RNA template there were two gene cassettes containing tandem connected exons, PER-6 and PER-7. One cassette carried 130A-222A-326C haplotype of SLC14A1 ORF and the other cassette carried 130G-222C-326T haplotype so that we could distinguish the two regions in the PacBio sequencing reads. By doing this we could also compare the artifact rates occurring at the two positions that were intensively investigated in this study.

As shown in S3 Fig in the revised manuscript, the indel rates in the 6-nucleotide region were much lower than that observed in the research subjects (Fig 4 in the revised manuscript), indicating RT-PCR artifacts can be ignored when analysing the transcription errors in this study.

Detailed information of the experiments, including the design, the results and summary as well as the discussion has been included in the revised manuscript.

Attachment Submitted filename: Response to Reviewers_PLOS ONE 20260724.docx

# Editor report: Decision Letter 2

**DOI:** 10.1371/journal.pone.0360437.r005

**Authors:** Santosh Patnaik

16 Sep 2026

Alterations of SLC14A1 mRNA structure are frequently detected in weak Jka presenting donors

PLOS One

Thank you for submitting your manuscript to PLOS ONE. After careful consideration, we feel that it has merit but does not fully meet PLOS ONE’s publication criteria as it currently stands. Therefore, we invite you to submit a revised version of the manuscript that addresses the points raised during the review process.

Thank you for submitting the revised manuscript with additional data and text to address the concerns raised in the previous review. I was unable to get any of the two referees who had examined the previous version of the manuscript to re-examine the revised manuscript. But I have gone through the manuscript changes and feel that they reasonably address the major concerns that were raised in the last review. However, I request you to edit the manuscript's abstract to add a statement effectively indicating that "additional experiments suggest that RT artifacts were unlikely the cause of these observations.

plosone@plos.org. When you're ready to submit your revision, log on to https://www.editorialmanager.com/pone/ and select the 'Submissions Needing Revision' folder to locate your manuscript file.

- A letter that responds to each point raised by the academic editor and reviewer(s). You should upload this letter as a separate file labeled 'Response to Reviewers'.
- A marked-up copy of your manuscript that highlights changes made to the original version. You should upload this as a separate file labeled 'Revised Manuscript with Track Changes'.
- An unmarked version of your revised paper without tracked changes. You should upload this as a separate file labeled 'Manuscript'.

If you would like to make changes to your financial disclosure, please include your updated statement in your cover letter. Guidelines for resubmitting your figure files are available below the reviewer comments at the end of this letter.

If applicable, we recommend that you deposit your laboratory protocols in protocols.io to enhance the reproducibility of your results. Protocols.io assigns your protocol its own identifier (DOI) so that it can be cited independently in the future. For instructions see: https://journals.plos.org/plosone/s/submission-guidelines#loc-laboratory-protocols. Additionally, PLOS One offers an option for publishing peer-reviewed Lab Protocol articles, which describe protocols hosted on protocols.io. Read more information on sharing protocols at https://plos.org/protocols?utm_medium=editorial-email&utm_source=authorletters&utm_campaign=protocols.

As the corresponding author, your ORCID iD is verified in the submission system and will appear in the published article. PLOS supports the use of ORCID, and we encourage all coauthors to register for an ORCID iD and use it as well. Please encourage your coauthors to verify their ORCID iD within the submission system before final acceptance, as unverified ORCID iDs will not appear in the published article. *Only*  the individual author can complete the verification step; PLOS staff *cannot*  verify ORCID iDs on behalf of authors.

We look forward to receiving your revised manuscript.

Kind regards,

Santosh K. Patnaik, MD, PhD

Academic Editor

PLOS One

Journal Requirements:

If the reviewer comments include a recommendation to cite specific previously published works, please review and evaluate these publications to determine whether they are relevant and should be cited. There is no requirement to cite these works unless the editor has indicated otherwise.

Please review your reference list to ensure that it is complete and correct. If you have cited papers that have been retracted, please include the rationale for doing so in the manuscript text, or remove these references and replace them with relevant current references. Any changes to the reference list should be mentioned in the rebuttal letter that accompanies your revised manuscript. If you need to cite a retracted article, indicate the article’s retracted status in the References list and also include a citation and full reference for the retraction notice.

[Note: HTML markup is below. Please do not edit.]

To ensure your figures meet our technical requirements, please review our figure guidelines: https://journals.plos.org/plosone/s/figures

You may also use PLOS’s free figure tool, NAAS, to help you prepare publication quality figures: https://journals.plos.org/plosone/s/figures#loc-tools-for-figure-preparation.

NAAS will assess whether your figures meet our technical requirements by comparing each figure against our figure specifications.

# Author comment: Author response to Decision Letter 3

**DOI:** 10.1371/journal.pone.0360437.r006

22 Sep 2026

Response to Editor’s comments

1. Thank you for submitting the revised manuscript with additional data and text to address the concerns raised in the previous review. I was unable to get any of the two referees who had examined the previous version of the manuscript to re-examine the revised manuscript. But I have gone through the manuscript changes and feel that they reasonably address the major concerns that were raised in the last review. However, I request you to edit the manuscript's abstract to add a statement effectively indicating that "additional experiments suggest that RT artifacts were unlikely the cause of these observations.

We thank Dr. Patnaik for taking time to examine our manuscript and greatly appreciate his approval of the revisions. According to his kind instruction, we have edited the abstract by adding "Technical artifacts were examined using synthesized RNA templates and the results suggested that artifacts generated in the reverse transcription and PCR amplification (RT-PCR) processes were unlikely the causes of these observations".

Attachment Submitted filename: Response to Reviewers_PLOS ONE 20260922.docx

# Editor report: Decision Letter 3

**DOI:** 10.1371/journal.pone.0360437.r007

**Authors:** Santosh Patnaik

27 Sep 2026

Alterations of SLC14A1 mRNA structure are frequently detected in weak Jka presenting donors

PONE-D-25-68594R3

Dear Dr. REN,

We’re pleased to inform you that your manuscript has been judged scientifically suitable for publication and will be formally accepted for publication once it meets all outstanding technical requirements.

Within one week, you’ll receive an e-mail detailing the required amendments. When these have been addressed, you’ll receive a formal acceptance letter and your manuscript will be scheduled for publication.

An invoice will be generated when your article is formally accepted. Please note, if your institution has a publishing partnership with PLOS and your article meets the relevant criteria, all or part of your publication costs will be covered. Please make sure your user information is up-to-date by logging into Editorial Manager at Editorial Manager® and clicking the ‘Update My Information' link at the top of the page. For questions related to billing, please contact billing support.

If your institution or institutions have a press office, please notify them about your upcoming paper to help maximize its impact. If they’ll be preparing press materials, please inform our press team as soon as possible -- no later than 48 hours after receiving the formal acceptance. Your manuscript will remain under strict press embargo until 2 pm Eastern Time on the date of publication. For more information, please contact onepress@plos.org.

Kind regards,

Santosh K. Patnaik, MD, PhD

Academic Editor

PLOS One

Additional Editor Comments (optional):

Reviewers' comments:

# Editor report: Acceptance letter

**DOI:** 10.1371/journal.pone.0360437.r008

**Authors:** Santosh Patnaik

PONE-D-25-68594R3

PLOS One

Dear Dr. REN,

I'm pleased to inform you that your manuscript has been deemed suitable for publication in PLOS One. Congratulations! Your manuscript is now being handed over to our production team.

At this stage, our production department will prepare your paper for publication. This includes ensuring the following:

* All references, tables, and figures are properly cited

* All relevant supporting information is included in the manuscript submission,

* There are no issues that prevent the paper from being properly typeset

You will receive further instructions from the production team, including instructions on how to review your proof when it is ready. Please keep in mind that we are working through a large volume of accepted articles, so please give us a few days to review your paper and let you know the next and final steps.

Lastly, if your institution or institutions have a press office, please let them know about your upcoming paper now to help maximize its impact. If they'll be preparing press materials, please inform our press team within the next 48 hours. Your manuscript will remain under strict press embargo until 2 pm Eastern Time on the date of publication. For more information, please contact onepress@plos.org.

You will receive an invoice from PLOS for your publication fee after your manuscript has reached the completed accept phase. If you receive an email requesting payment before acceptance or for any other service, this may be a phishing scheme. Learn how to identify phishing emails and protect your accounts at https://explore.plos.org/phishing.

If we can help with anything else, please email us at customercare@plos.org.

Thank you for submitting your work to PLOS One and supporting open access.

Kind regards,

PLOS One Editorial Office Staff

on behalf of

Dr. Santosh K. Patnaik

Academic Editor

PLOS One
